# *Ex vivo* modeling of lung tissue resident antimicrobial responses

**DOI:** 10.1128/mbio.00056-26

**Published:** 2026-04-16

**Authors:** Hélèna Choltus, Niccolo Bianchi, Julien Prados, Nelli Heikkila, Wolfram Karenovics, Veronique Serre Beinier, Yannick Nissille, Nina Leitner, Benoit Bédat, Fedor Bezrukov, Beryl Mazel-Sanchez, Virginie Prendki, Alexander Lobrinus, Christiane Eberhardt, Simone Becattini, Mirco Schmolke

**Affiliations:** 1 Department of Microbiology and Molecular Medicine, Medical Faculty, University of Geneva 27212, Geneva, Switzerland; 2 Department of Pathology and Immunology, Medical Faculty, University of Geneva 27212, Geneva, Switzerland; 3 Bioinformatics Core Facility, Medical Faculty, University of Geneva 27212, Geneva, Switzerland; 4 Department of Pediatrics, Gynecology and Obstetrics, University Hospital of Geneva (HUG), Geneva, Switzerland; 5 Thoracic and Endocrine Surgery Division, University Hospitals Geneva (HUG), Geneva, Switzerland; 6 Department of Surgery, Medical Faculty, University of Geneva 27212, Geneva, Switzerland; 7 National Reference Center for Influenza (CNRI), University Hospitals Geneva (HUG), Geneva, Switzerland; 8 Department of Rehabilitation and Geriatrics, Geneva University Hospitals 27230 , Geneva, Switzerland; 9 Institute of Pathology, Lausanne University Hospital (CHUV) 30635, Lausanne, Switzerland; 10 Department of Clinical Pathology, University Hospital Geneva, Geneva, Switzerland; 11 Center for Vaccinology, Medical Faculty, University of Geneva 27212, Geneva, Switzerland; 12 Geneva Center for Inflammation Research, Medical Faculty, University of Geneva 27212, Geneva, Switzerland; McMaster University, Hamilton, Ontario, Canada

**Keywords:** PCLS, precision cut lung slices, *ex vivo *model, preclinical model, antimicrobial, innate immunity, influenza virus, *Streptococcus pneumoniae*, 3R, infectious disease, interferon, interleukin

## Abstract

Tissue resident host responses to microbial infections in the respiratory tract are highly dynamic in space and time and rely on the interaction of a multitude of cell types. To model these multicellular responses reliably in cell culture, we compare here the global transcriptional antimicrobial response to infection with influenza A virus (IAV) in precision cut lung slices (PCLS), volume-defined organ discs largely maintaining the cellular composition and 3D architecture of the donor lung. To permit a fair comparison of host responses in an isogenic background, we first challenged mice *in vivo* and murine PCLS (mPCLS) and assessed host transcriptomic changes by unbiased RNA-sequencing. While core antiviral responses overlapped substantially, mPCLS lacked certain features—such as type II interferon expression—likely due to the absence of infiltrating immune cell responses. Importantly, when expanding our findings to immune-experienced human precision cut lung slices (hPCLS), we find a much broader antiviral response after IAV challenge, including types I, II, and III interferons, suggesting the presence of responsive tissue resident lymphocytes. To prove the specificity of this response, we infected hPCLS with *Streptococcus pneumoniae. Ex vivo* tissues responded with a distinct proinflammatory gene profile including IL1A, IL1B, and IL17 expression. Blocking of IL-1β signaling partially inhibited the proinflammatory response, suggesting cellular cross talk and a complex and specific antimicrobial reaction in this *ex vivo* model. In conclusion, a diversified tissue resident immune cell compartment distinguishes the human *ex vivo* model, making it an ideal system for microbiological and immunological research.

**IMPORTANCE:**

Pathogen interactions with the lungs are very dynamic processes. In biomedical research, it is paramount to model these processes in the laboratory as accurately as possible. Influenza A virus has been extensively studied in epithelial cell culture models, including advanced organoids and organ-on-a-chip systems. Here, we use *ex vivo* cultured precision cut lung slices (PCLS) and generate transcriptomic data to assess the global tissue resident host response to viral and bacterial challenges. Our data show (i) that murine PCLS faithfully reflect core responses to viral infection, while missing proinflammatory responses linked to infiltrating immune cells and (ii) that human PCLS show a highly diversified tissue resident immune response to viral infection due to previous exposures of the host to this pathogen. These responses are clearly distinct from antibacterial gene profiles. Our data advertise PCLS as a complex and realistic model to study tissue resident immune responses to microbes in a human system.

## INTRODUCTION

Respiratory infections with viral or bacterial pathogens are the fifth leading cause of death worldwide and the leading cause of death in low-income countries (excluding COVID-19) according to 2021 World Health Organization estimations (1). Continuous exposure of lung mucosa to the inhaled air creates a primary entry point for airborne viral and bacterial pathogens. Among these, influenza viruses alone are responsible for more than half a million deaths annually, often complicated by secondary bacterial pneumonia, mostly with *Streptococcus pneumoniae* or *Staphylococcus aureus* (2).

The first line of defense against respiratory infections is orchestrated by airway and alveolar epithelial cells, which in concert with tissue-resident immune cells initiate antimicrobial and inflammatory responses that restrict pathogen spread. However, modeling the complexity of antimicrobial host responses of the respiratory tract *in vitro* remains challenging, in part since the human lung consists of more than fifty cell types (3). 2D cell culture models do not reflect this cellular diversity and the complex spatial 3D organization of respiratory tissue that is critical for an accurate recapitulation of the lung host defense.

Precision-cut lung slices (PCLS) have emerged as a powerful *ex vivo* model for pre-clinical lung research. These volume-defined discs of human or animal lung tissue origin are cultured *ex vivo* and bridge conventional cell culture models and *in vivo* systems (4, 5). Importantly, they preserve the cellular composition and the native architecture of airway and alveolar spaces, allowing the study of host–pathogen interactions within a near-physiological tissue context (6).

In recent years, PCLS derived from both human and murine lungs (even passing by swine, bovine, or caprine lungs in veterinary research) have been increasingly used to model infections with respiratory viruses and bacteria (7–10). Since PCLS are disconnected from the systemic circulation, they provide a unique opportunity to interrogate tissue-resident immune responses in isolation, without the confounding effects of recruited circulating immune cells or systemic inflammatory responses. Today, comparative analyses of in-tissue antimicrobial responses across species and between *ex vivo* and *in vivo* systems remain limited (11). Understanding how *ex vivo* murine and human lung tissues differ from early *in vivo* innate immune responses to viral and bacterial challenge is crucial for the translation of findings from animal models to human biology. PCLS could fill this knowledge gap and at the same time serve as a 3R-compatible tissue culture model.

Here, we compare the tissue-resident host response to viral and bacterial challenge in murine and human PCLS and benchmark these *ex vivo* responses against transcriptomic data derived from *in vivo* infected lung tissues. This integrated approach supports the translational relevance of PCLS as a model for studying respiratory infections.

## RESULTS

The antiviral host response to influenza A viruses (IAVs) in the respiratory tract is an extraordinarily dynamic process initiated by infected epithelial cells and surveilling tissue resident immune cells. This first line of defense can be decisive for the outcome of infection. It establishes an antiviral state in the respiratory tissue, most importantly induced by cytokines of the interferon family. It further attracts immune cells from the blood and the interstitial space into the lung to limit and clear viral infection.

**Fig 1:**
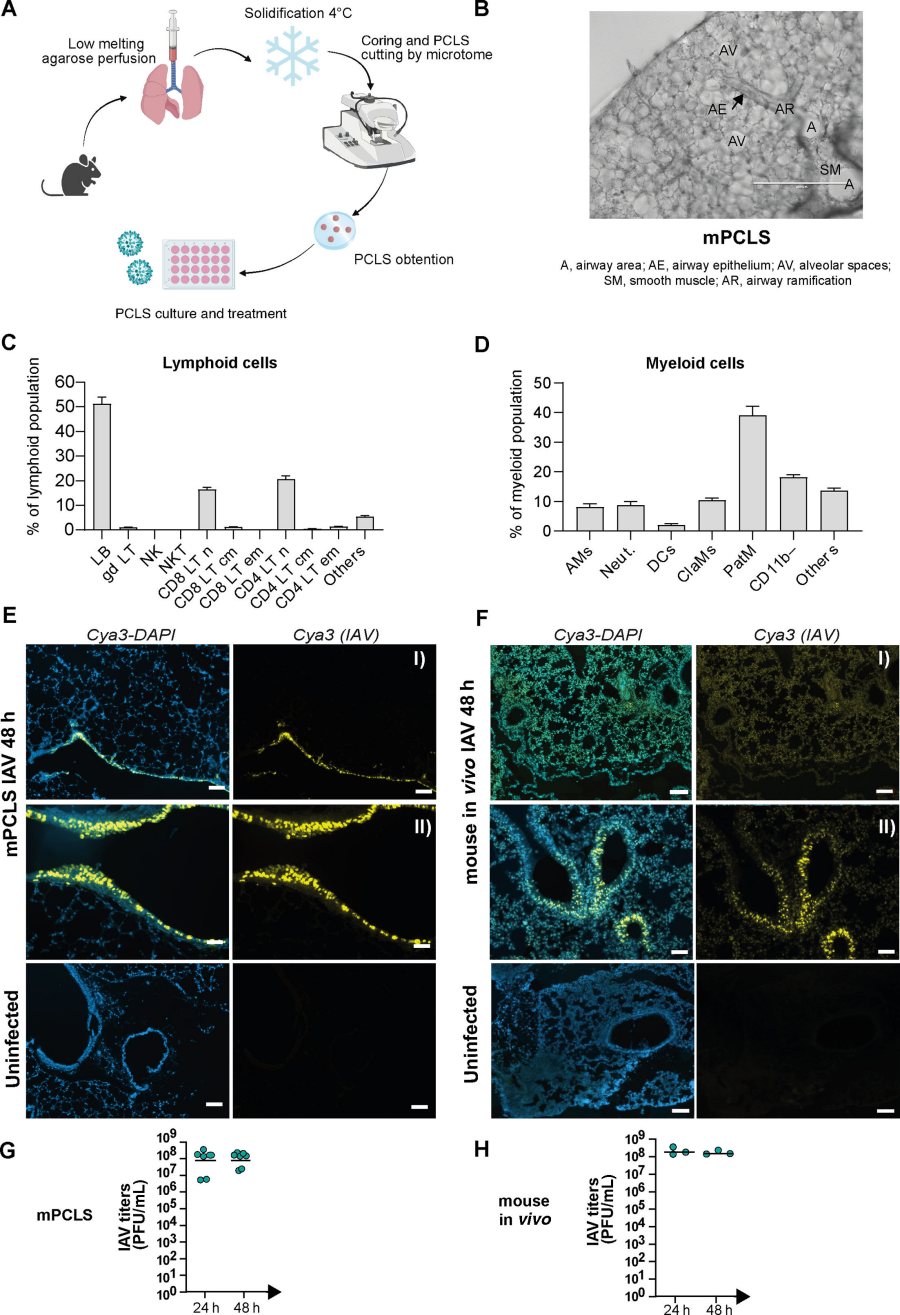
Modeling the early host response against Influenza A virus in murine precision-cut lung slices (mPCLS). (**A**) Schematic of PCLS generation: lungs are perfused with a low melting agarose solution to preserve the 3D lung architecture. After solidification at 4°C, lungs are cored into regular cylinders and processed to slicing using a microtome. PCLS are then kept in culture medium at 37°C. (**B**) Brightfield picture of an untreated murine PCLS. Scale bar represents 400µm. (**C and D**) Untreated PCLS were enzymatically digested one day after slicing to characterize baseline composition of lymphoid (**C**) and myeloid (**D**) immune cells. Results are presented in % of total lymphoid population and in % of total myeloid population (*N* = 3). Lymphoid cells: LB, lymphocytes B; gdLT, γδ T lymphocytes; NK, natural killer cells; NKT, natural killer T cells; LT, lymphocyte T; n, naïve; cm, central memory; em; effector memory. Myeloid cells: AMs, alveolar macrophages; Neut, neutrophils; DCs, dendritic cells; ClaMs, classical monocytes; PatMs, patrolling monocytes. Mice and mPCLS were respectively infected with 10^4^/10^5^ PFU of IAV (A/Netherlands/602/2009/H1N1) for 24 or 48 h. (**E and F**) *In situ* RNA hybridization of viral nucleoprotein (cyanine 3) was used to visualize influenza A virus (IAV) at 48 hpi in mPCLS sections (**E**) and *in vivo* in mouse lungs (**F**). Confocal observation was done at ×100 (top pictures IAV [**I**] and uninfected, size bar represents 100 µm) or at ×200 (bottom pictures IAV [II], size bar represents 50 µm). (**G and H**) Titers were measured by plaque assay on mPCLS (**G**, *N* = 4, duplicates) and on *in vivo* infected lung (**H**, *N* = 3) at 24 and 48 hpi.

To delineate early tissue resident antiviral responses from those after immune cell infiltration, we turned to the murine PCLS (mPCLS) model (Fig. 1A). Brightfield microscopy confirmed the maintenance of typical lung tissue architecture after tissue slicing (Fig. 1B). mPCLS were cultured for several days without signs of tissue disintegration (Fig. S1A and B). The immune cell compartment of mPCLS was predominantly characterized by naïve CD4 T cells and B cells in the lymphoid panel (Fig. 1C, gating strategy in Fig. S2A) and monocytes (classical and non-classical) in the myeloid panel (Fig. 1D, gating strategy in Fig. S2B). As compared to published *in vivo* data (12), mPCLS show a slightly higher proportion of B lymphocytes vs T lymphocytes and a generally lower representation of myeloid cells, suggesting that the latter might be partially excluded in the process of PCLS preparation.

The porous nature of this *ex vivo* model permitted IAV infection with a clinically relevant H1N1 strain influenza A/Netherlands/602/2009 (H1N1) throughout the tissue as demonstrated by specific RNAscope staining of RNA in mid-sections 48 h post-infection (Fig. 1E). We did, however, observe a preference for infection in larger airways. A similar distribution of RNA signal was found in *in vivo* infected lung tissue 48 h post-infection (Fig. 1F). In line with this, viral titers *ex vivo* and *in vivo* reached comparable levels 48 h post-infection (Fig. 1G and H).

**Fig 2:**
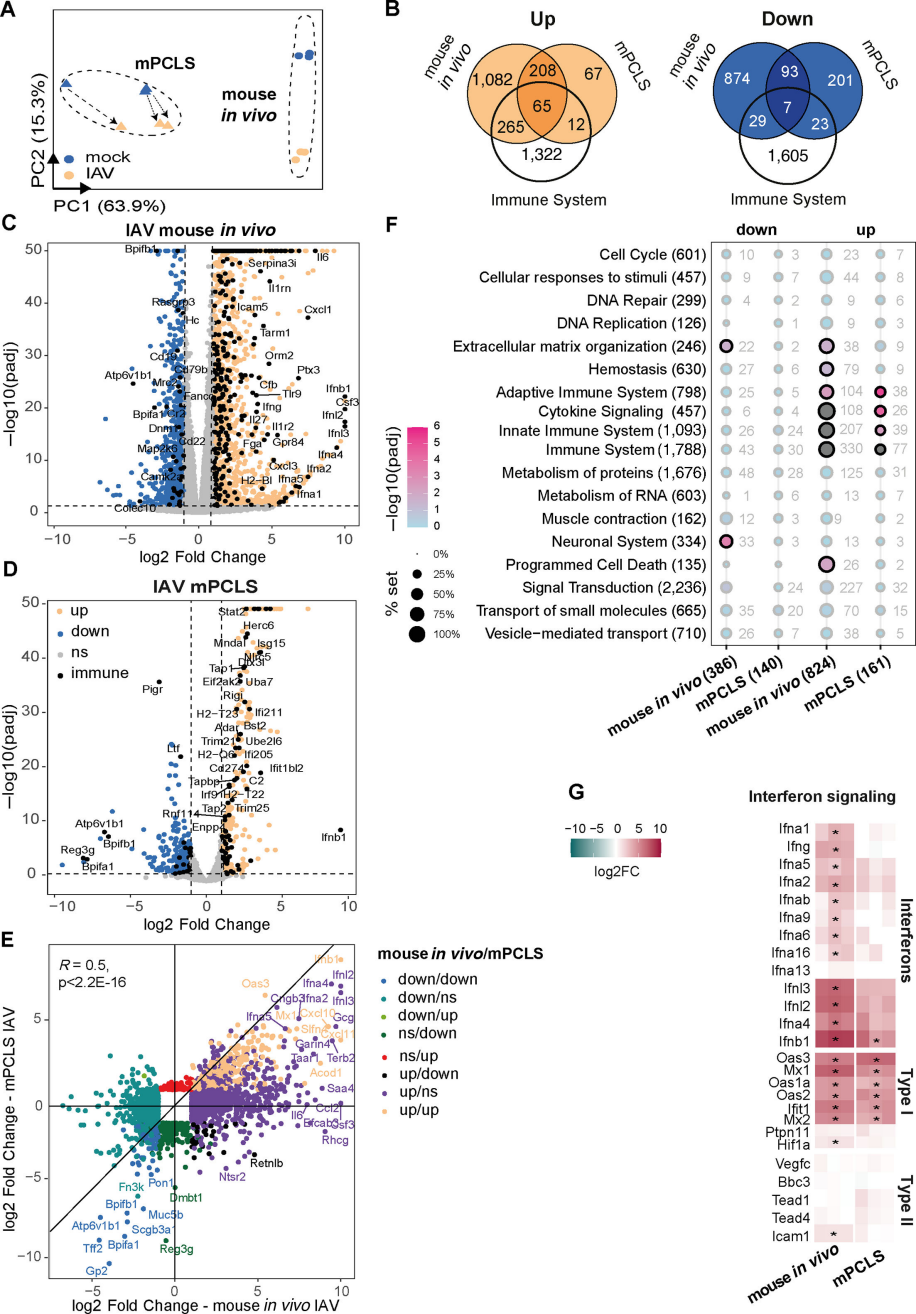
Comparative transcriptomic analysis of the early host response to influenza A virus (IAV) in murine lung *in vivo* and *ex vivo*. Mice and murine PCLS (mPCLS) were respectively infected with 10^4^/10^5^ PFU of IAV (A/Netherlands/602/2009/H1N1) for 48 h. Tissue was processed to bulk RNA sequencing and host response was analyzed on R using DESeq2 package. (**A**) Principal component analysis illustrates the transcriptional response of mock-treated (blue) and IAV-infected lung tissue (orange) *in vivo* and in mPCLS. Arrows indicate the pairs of mock and infected PCLS from the three independent experiments. (**B**) Venn diagrams of the commonly upregulated (orange) or downregulated (blue) genes in mouse *in vivo* and mPCLS infected with IAV. The white circle indicates the genes related to immune system pathways (using Reactome annotations). (**C and D**) Volcano plot showing differentially expressed genes between IAV-infected and mock samples in mouse *in vivo* (**C**) and in mPCLS (**D**). Log2 fold change induction is indicated in the *x*-axis, −log10(*p*-adjusted) in the *y*-axis. Genes have been represented in orange (upregulated), blue (downregulated), and gray (not significant). Genes associated with immune system pathways are depicted in black. (**E**) Scatter plot comparing the genes regulated by IAV in mouse *in vivo* (log2 fold change in *x*-axis) and in mPCLS (log2 fold change in *y*-axis). Correlation is indicated with *R* Pearson coefficient (*R* = 0.5, *P* < 2.2^−16^). Color legend indicates the direction for mouse *in vivo*/mPCLS genes regulated. (**F**) Pathway enrichment analysis was conducted using hypergeometric tests against Reactome.org database. −log10 (*P*-adjusted) is given by a color scale. Coverage percentage of the pathway set is indicated by the size of the dot. Pathways are indicated with the total number of genes in brackets, and the number of genes significantly regulated is indicated next to the dot. Significantly enriched pathways are indicated by a black circle. (**G**) Heatmap illustrates the regulation of interferon signaling genes by IAV 48 hpi in mouse *in vivo* and in mPCLS. Genes are represented by the log2 fold change (pink: positive log2FC, blue: negative log2FC); * indicates if the gene is significantly regulated by IAV infection.

Using a bulk RNA-sequencing (RNA-seq) approach, we compared transcriptional responses in IAV-infected lung tissue *in vivo* and *ex vivo*. Not surprisingly, the principle component analysis (PCA) separated host responses clearly between the two models (along PC1), while the IAV induced a parallel shift of the global transcriptome along PC2 in the two systems, which was less prominent especially in the *ex vivo* model (Fig. 2A). Accordingly, we found substantially more differentially upregulated and downregulated genes (DEG) *in vivo* than in mPCLS (Fig. 2B through D). Approximately 20% of the upregulated protein coding DEG were classified as “immune system” related in the Reactome database (13) (Fig. 2B). Notably, IAV-induced DEG in mPCLS were largely found upregulated *in vivo* (Fig. 2B). The two gene sets correlated with an *R*^2^: 0.5 (Fig. 2E). IAV infection has been reported to induce a general host transcription shutoff, resulting in widespread and untargeted downregulation of host gene expression (14). Accordingly, the overlap of downregulated genes between the two models was limited, and gene enrichment analysis only provided significance for extracellular matrix (ECM) organization and neuronal system–related processes (Fig. 2F), including ion channel activity, which has previously been described following influenza infection in mice (15, 16).

An analysis of transcription factor binding sites in promoters of upregulated genes in mPCLS indicated that IRF3 and STAT2 are mostly responsible for driving the *ex vivo* response, while *in vivo* NFκB, STAT5B, NOD2, and IRF3 were the most significantly enriched transcription factors (Fig. S3A and B) (17–19), suggesting a more prominent inflammatory component *in vivo*. Comparative Reactome analysis of mPCLS and *in vivo* mouse responses to IAV revealed a strong enrichment in immune system including cytokine signaling, innate and adaptive immunity in both models (Fig. 2F). While for mPCLS, the response was restricted to immune system activation, we found *in vivo* additionally an enrichment in hemostasis, extracellular matrix organization, and programmed cell death among the DEG (Fig. S3C through E). To complement the deficiency of a pronounced inflammatory response due to the lack of infiltrating immune cells, we cocultured mPCLS *ex vivo* with heterologous bone marrow-derived cells (BMC, Fig. S4A and B) or bone marrow-derived macrophages (BMDM, Fig. S4C and D). While the coculture with BMC had no effect on host response and viral titers, addition of BMDM resulted in increased IL-6 mRNA levels and a reduction of viral titers by more than one order of magnitude (Fig. S4D). These data suggest that a functional complementation by addition of immune cells in *trans* could enrich the PCLS model.

**Fig 3:**
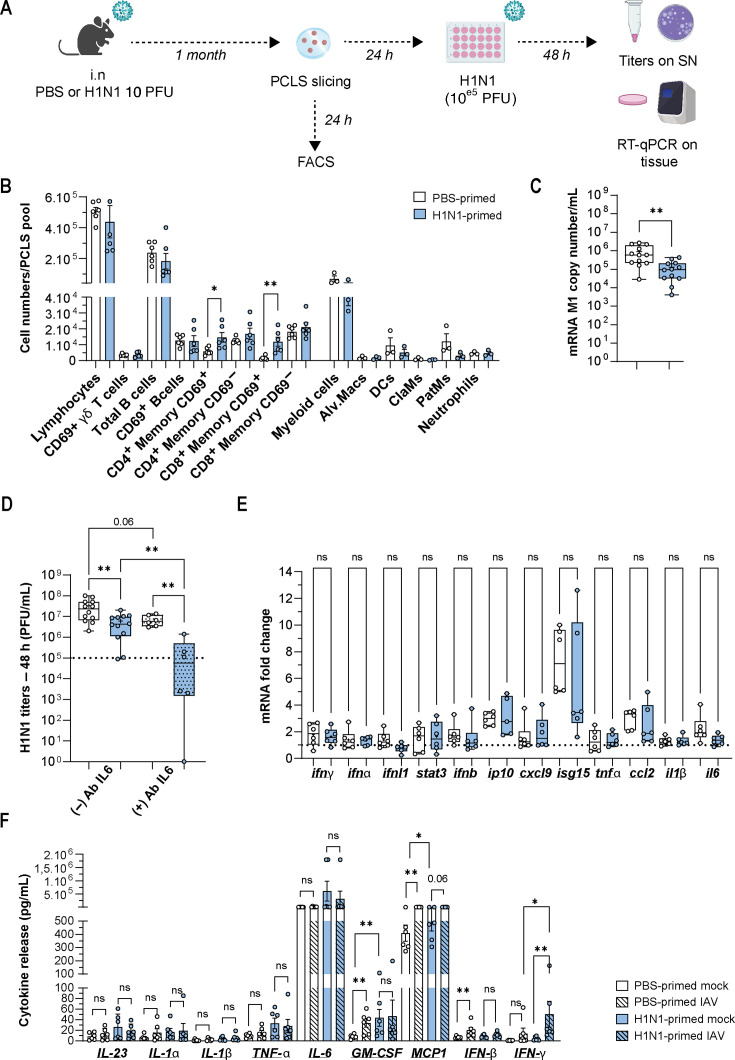
Characterization of the innate immune response in influenza A virus (IAV)-primed murine PCLS (mPCLS). (**A**) Mice were intranasally primed with phosphate-buffered saline (PBS) or 10 PFU of A/Netherlands/602/2009/H1N1 (*N* = 3–6, duplicates). One month later, mice were sacrificed, and mPCLS were generated and homologously re-infected with 10^5^ PFU of IAV for 48 h. Supernatants and tissues were collected for titers and RNA extraction. (**B**) Flow cytometry was conducted on untreated mPCLS from PBS or IAV-primed mice. Lymphoid and myeloid cell populations were quantified. (Alv.Macs: alveolar macrophages, DCs: dendritic cells, ClaMs: classical Ly6 high monocytes, PatMs: patrolling Ly6 low monocytes). (**C**) Viral replication was measured at 48 hpi by reverse transcription-quantitative PCR (RT-qPCR) on influenza matrix M1 gene on IAV-infected PCLS from PBS and IAV-primed mice. (**D**) Viral titers 48 hpi were measured by plaque assay. (**E**) RT-qPCR on inflammatory genes was performed at 48 hpi. Fold change over mock is represented. (**F**) Cytokine release in the supernatants was quantified using a multiplex assay at 48 hpi. Statistical analysis was done with a non-parametric unpaired *t*-test (*N* = 3–6). PBS-primed mPCLS are represented in white boxes and IAV-primed mPCLS in blue boxes (**: *P* < 0.01, *: *P* < 0.05).

A common denominator of the antiviral response in human and murine hosts is the interferon response (20). *Ex vivo* type I and III interferon genes were generally upregulated to a much lower extent than *in vivo*, with IFNB1 being the most highly induced type I interferon gene (Fig. 2G). Accordingly, we found upregulation of typical ISGs like MX1, OAS1, or IFIT1 (among others). Notably, mPCLS were completely devoid of a type II interferon response. Accordingly, ICAM1, an experimentally confirmed type II IFN-dependent gene (21), was not upregulated in mPCLS after IAV challenge, but type I-/type III-dependent genes were (Fig. 2G). IFNγ is almost exclusively produced by lymphoid cells (NK, NKT, effector/memory CD4^+^, CD8^+^, and γδ T lymphocytes). These cells were largely absent in mPCLS (Fig. 1C), possibly explaining the lack of IFNγ in response to IAV infection. Both our PCLS donor mice and the *in vivo* infected animals were immunologically naïve to IAV infection, explaining the lack of tissue resident memory cells. To simulate tissue responses of immune-experienced mice, we generated mPCLS from IAV (A/Netherlands/602/2009 H1N1) primed mice 1 month after primoinfection (Fig. 3A) and challenged these immune-experienced mPCLS with the same virus strain *ex vivo*. FACS revealed an increase in activated memory CD4 and CD8 T lymphocytes in H1N1-primed PCLS, but no enrichment in myeloid cells was observed (Fig. 3B and gating strategy in Fig. S2B and C). Viral replication was reduced in *ex vivo* tissues from IAV-primed mice as confirmed by quantitative PCR (qPCR) for viral RNA and by titration of viral supernatants (Fig. 3C and D). Surprisingly, this did not result in a generally heightened transcriptional host response in IAV-primed murine lung tissues (Fig. 3E) or in higher secreted cytokine levels (Fig. 3F), with the exception of modestly increased IFNγ release by IAV-infected H1N1-primed PCLS. Our data hence suggest an increase in viral defense with improved control of viral replication 1 month after priming, which does not generally depend on an upregulation of innate immune genes, nor seems to promote it upon *ex vivo* reinfection of tissues.

On average, a human is exposed every 5–10 years to a new influenza virus (22). These repeated infections lead to an accumulation of tissue-resident memory T cells in adults. In elderly influenza-specific _trm_T cells decline with advanced age in lung tissue, which could in part contribute to the reduced antiviral response (23). Since the elderly form a major population at risk for severe IAV infection, we asked if PCLS from an immune-experienced elderly human donor would provide a more realistic model of tissue resident immune responses against IAV.

**Fig 4:**
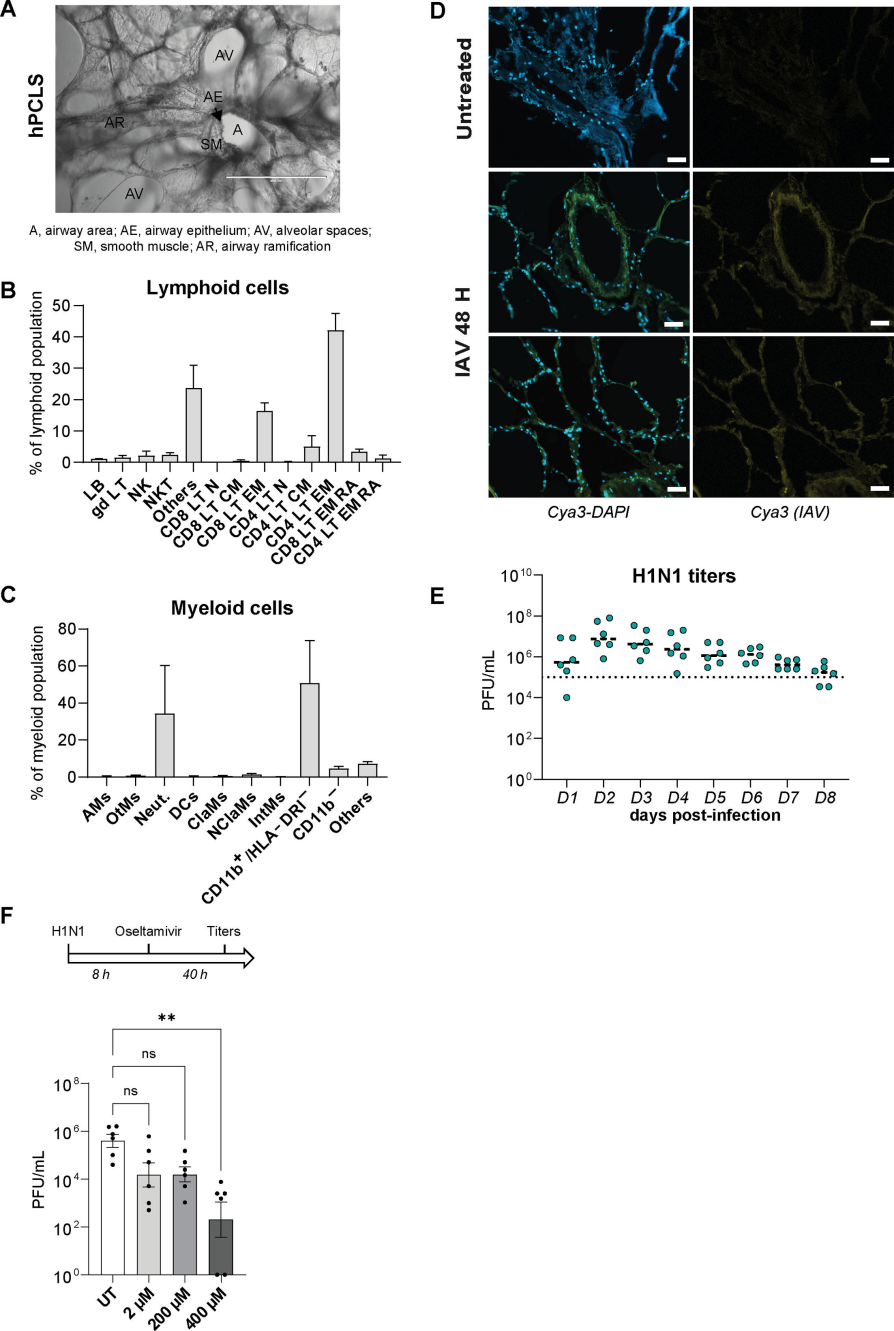
Antimicrobial host response to viral challenge in the human PCLS model. (**A**) Brightfield picture of an untreated human PCLS in culture (magnification ×100, size bar represents 400 µm). (**B and C**) Untreated PCLS from three patients were enzymatically digested one day after slicing to describe the baseline composition of lymphoid (**B**) and myeloid (**C**) immune cells. Results are presented in % of total lymphoid population and in % of total myeloid population. Lymphoid cells: LB, lymphocytes B; gdLT, γδ T lymphocytes; NK, natural killer cells; NKT, natural killer T cells; LT, lymphocyte T; n, naïve; cm, central memory; em, effector memory; emra, effector memory CD45RA^+^. Myeloid cells: AMs, alveolar macrophages; OtMs, other macrophages; Neut, neutrophils; DCs, dendritic cells; ClaMs, classical monocytes; NClaMs, non-classical monocytes; IntMs, intermediate monocytes. (**D**) *In situ* RNA hybridization of viral nucleoprotein (cyanine 3) was used to visualize IAV at 48 hpi in hPCLS sections. Magnification ×200. Size bar represents 50 µm. (**E**) hPCLS were infected with 10^5^ PFU of IAV (A/Netherlands/602/2009/H1N1) for 8 days; viral titers were measured daily by plaque assay (three patients in duplicates). (**F**) Antiviral drug efficacy was tested in hPCLS after H1N1 infection. Oseltamivir carboxylate was added 8 hpi at different doses (2, 200, and 400 µM). Supernatants were collected 48 hpi to process to virus titration (*N* = 3 patients, duplicates). Statistical analysis was performed using a one-way analysis of variance (**: *P* < 0.01).

PCLS were cut from histologically healthy bystander tissue of tumor resections (for patient cohort data, see Table S1) and maintained typical 3D architecture of human lung tissue (Fig. 4A). Notably, airspaces were much larger than those found in mPCLS (Fig. 1B). *Ex vivo* human PCLS (hPCLS) were cultured for several days without signs of cell death or inflammation comparable to mPCLS (Fig. S1C and D). In contrast to murine tissues, T lymphocytes were the dominant lymphoid cell population in hPCLS among these more than 50% effector memory CD4 and CD8 T cells (Fig. 4B and gating strategy in Fig. S2D). Additionally, we found low percentages of NK and NKT cells, absent in *ex vivo* mouse tissues. In the myeloid panel, monocytic myeloid-derived suppressor cells (24) and neutrophils were dominant (Fig. 4C and gating strategy in Fig. S2E).

As for mPCLS, IAV penetrated the entire tissue (Fig. 4D) and resulted in robust viral titers 48 hpi (Fig. 4E). We found little donor to donor variation in the replication data, despite using tissues from different anatomical locations of the lung and diverse underlying disease states (Table S1), supporting the robustness of this model system for microbiological research. To further test the applicability of the PCLS model to antiviral research, we tested different doses of oseltamivir carboxylate, a standard of care neuraminidase inhibitor used in the treatment of IAV, typically administered within 48 h of symptom onset (25). Following oral application, oseltamivir concentrations found in patients’ fluids (sinuses, plasma, and middle ear) range from 200 to 400 µM. In our hands, 1-log_10_ IAV titer decrease was observed with 2 and 200 µM, and a significant 4-log_10_ reduction was obtained with 400 µM at 48 hpi in IAV-infected hPCLS (Fig. 4F), suggesting that hPCLS realistically mirror the therapeutic *in vivo* window of oseltamivir *ex vivo*.

**Fig 5:**
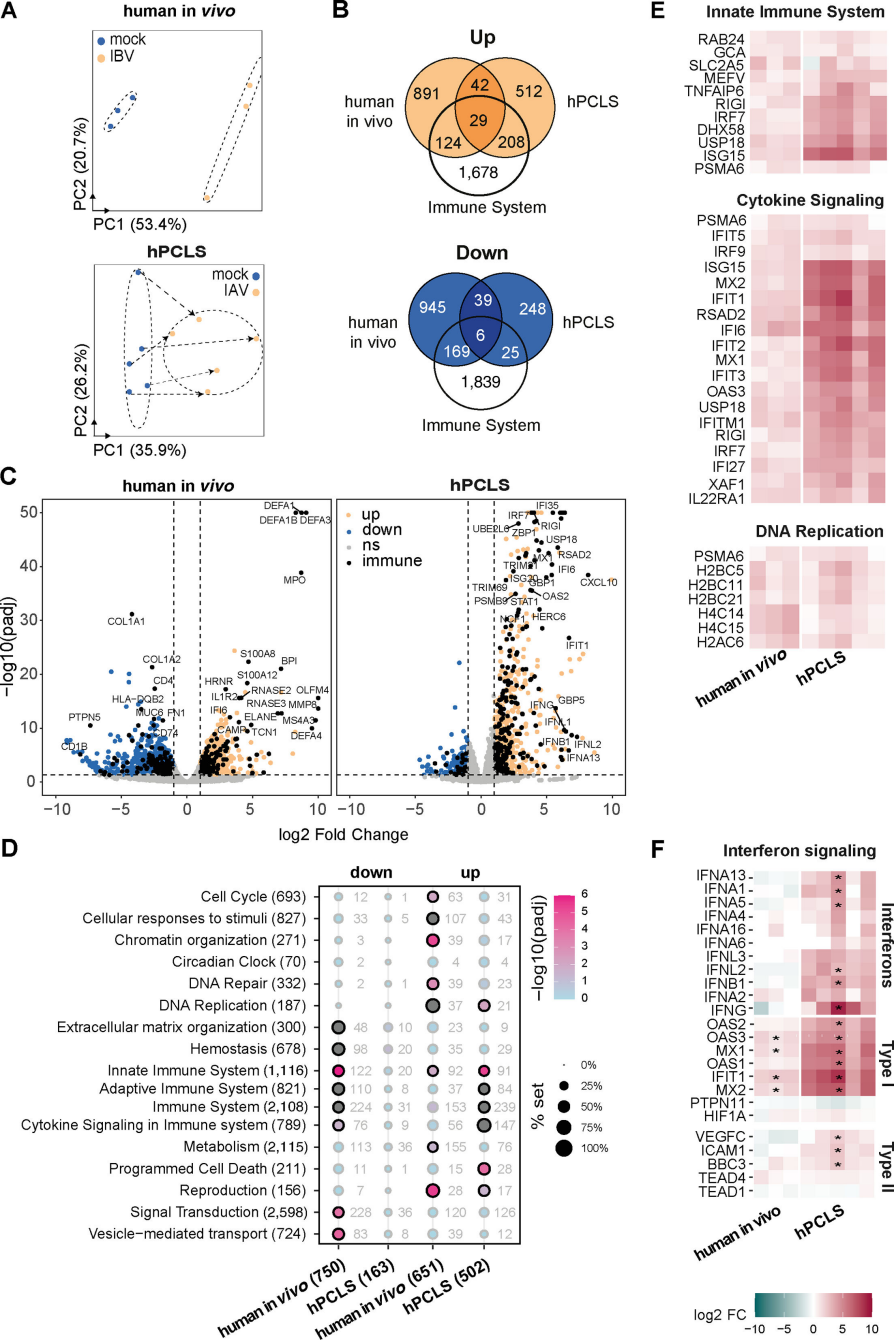
Antimicrobial host response to viral challenge in the human PCLS (hPCLS) model versus *in vivo* human lungs. Human PCLS from five patients were infected with 10^5^ PFU of IAV (A/Netherlands/602/2009/H1N1) for 48 h. In parallel, lung biopsies from a naturally influenza-infected patient *in vivo* (triplicates from three different lobes) were compared to unprocessed lung samples from three uninfected patients. Bulk RNA-sequencing was performed on these samples, and analysis was conducted using DESeq2 package on R software. (**A**) Principal component analysis illustrates the global transcriptomics response of control (blue) and influenza-infected (orange) samples in human *in vivo* (left) and in hPCLS (right). (**B**) Venn diagrams of the commonly upregulated (orange) or downregulated (blue) genes in human *in vivo* and hPCLS after influenza infection. The white circle indicates the genes related to immune system pathways. (**C**) Volcano plot showing differentially expressed genes between influenza-infected and mock samples in human *in vivo* (left) and in hPCLS (right). Log2 fold change induction is indicated in the *x*-axis, −log10(*P*-adjusted) in the *y*-axis. Genes have been represented in orange (upregulated), blue (downregulated), and gray (not significant). Genes associated with immune system pathways are illustrated in black. (**D**) Pathway enrichment analysis was conducted using the Reactome database. −log10 (*P*-adjusted) is given by a color scale. Coverage percentage of the pathway set is indicated by the size of the dot. Pathways are indicated with the total number of genes in brackets, and the numbers of genes significantly regulated are indicated next to the dot. Pathways significantly enriched are surrounded by a black circle. (**E**) Heatmaps indicate the commonly up genes in humans *in vivo* and in hPCLS after influenza infection for innate immune system, cytokine signaling, and DNA replication pathways. Genes are ranked by the log2 fold change (upregulated, pink; downregulated, blue). (**F**) Heatmap of genes associated with interferon signaling. The significance of regulation by IAV in human *in vivo* and in hPCLS is indicated by an *.

Next, we assessed the host transcriptional response of hPCLS to IAV infection. The PCA clearly separated mock-treated from IAV-infected hPCLS 48 hpi. This is similar to data obtained from an influenza virus RNA-positive lung autopsy tissue from a patient who died shortly after an influenza B virus diagnosis when compared to freshly isolated healthy lung tissue material (Fig. 5A). In contrast to the mouse model, comparable numbers of DEGs were upregulated in hPCLS and human *in vivo* infected lung tissue. However, at the protein-coding gene level, we found only a small overlap of the upregulated DEG of hPCLS with the *in vivo* tissues (Fig. 5B and C). This could be the consequence of several confounding effects: comparison of non-isogenic hosts, comparison of an influenza A virus vs an influenza B virus infection, different sequencing protocols (standard Illumina RNA-seq vs exon enrichment for *in vivo*), and, most importantly, differences in the time point post-infection. Nevertheless, the Reactome analysis provided substantial overlap in upregulated gene modules related to innate immune response, cytokine signaling, and DNA replication, the latter being absent in murine samples (Fig. 5D and E). On the gene level, the intersection of upregulated genes that contained known ISGs like MX2, IFI6, IFIT1, IFIT5, IFIT3, IFI27, IRF27, IRF9, ISG15, and IFITM1 was found. IAV infection robustly induced types I, II, and III interferons in hPCLS. Upregulation of IFNγ and IFNγ-induced genes after IAV infection (Fig. 5F) is of interest in this model since it confirms the activation and functionality of tissue resident lymphoid cells. We would like to stress that these preliminary *in vivo* findings would require confirmation from larger post-mortem cohorts.

**Fig 6:**
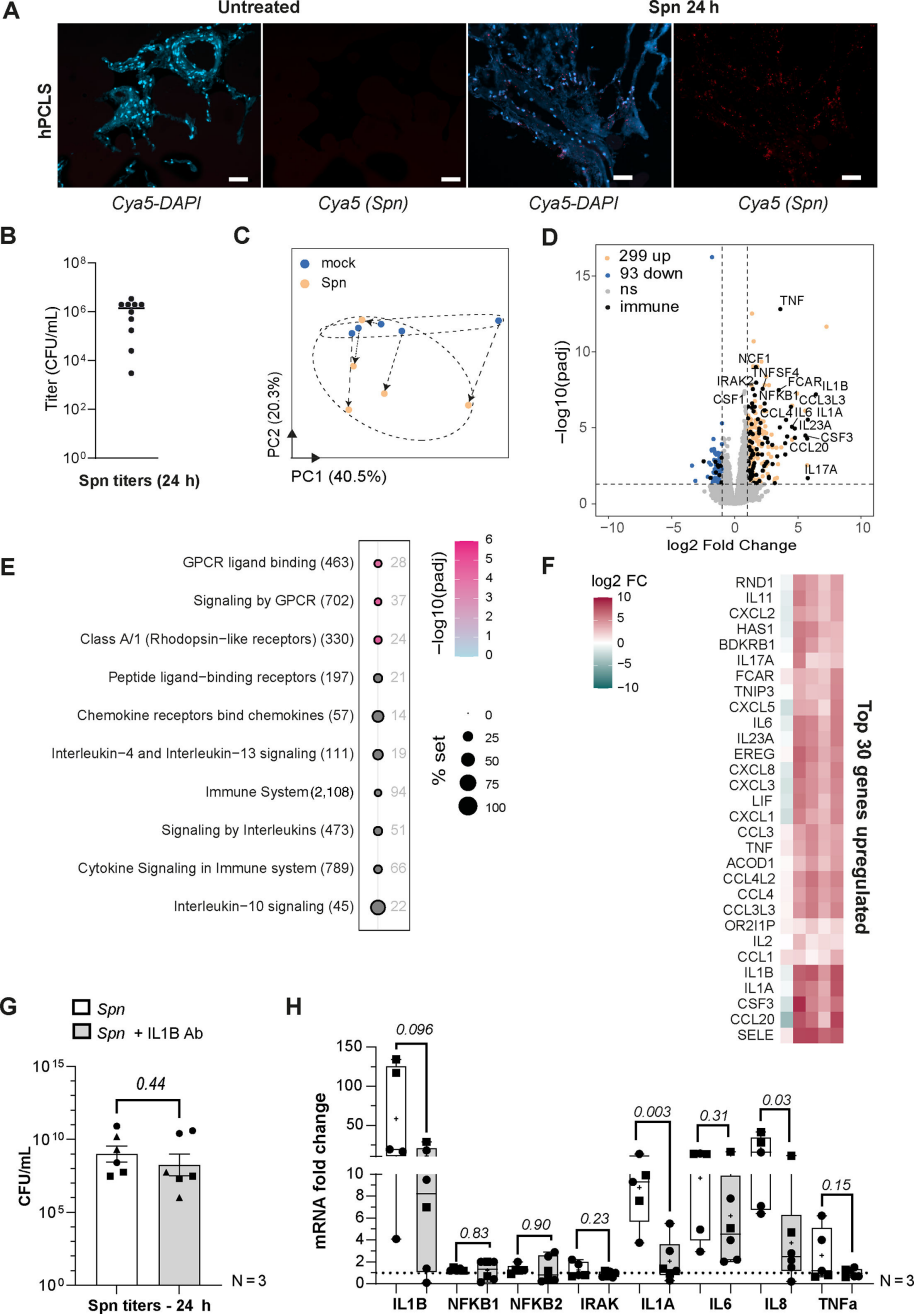
hPCLS can reproduce a classical antibacterial response toward a bacterial challenge. Human PCLS (hPCLS) from five patients were infected with 10^3^ CFU of *Streptococcus pneumoniae* (Klein Chester ATCC 6303, serotype 3) for 24 h. Bioinformatic analysis was done using DESeq2 with R programming. (**A**) *In situ* RNA hybridization of dnaA (Cyanine 5) was used to visualize *Spn* at 24 hpi in hPCLS sections. Magnification ×100. Size bar is representing 100 µm. Mock samples are uninfected hPCLS. (**B**) Bacterial titers from tissue homogenates of PCLS were measured at 24 hpi (*N* = 5, duplicates). (**C**) Principal component analysis illustrates the global transcriptomics response of mock-treated (blue) and Spn-infected 24 hpi (orange) hPCLS. Arrows indicate the corresponding infected sample for each patient mock sample. (**D**) Volcano plot showing differentially expressed genes between Spn-infected and mock-treated samples. Log2 fold change induction is indicated in the *x*-axis, *P*-adjusted in the *y*-axis. Genes have been represented in: orange (upregulated), blue (downregulated), and gray (not significant). Genes associated with immune system pathways are represented in black. (**E**) Pathway enrichment analysis was conducted using the Reactome database (hypergeometric test). The top 10 pathways upregulated by *Spn* infection are shown. −Log10 (*P*-adjusted) is given by a color scale. Coverage percentage of the pathway set is indicated by the size of the dot. Pathways are indicated with the total number of genes in brackets and the numbers of genes significantly regulated are indicated next to the dot. (**F**) Heatmaps of the top 30 genes significantly upregulated (pink) and downregulated (blue) by Spn in hPCLS from 5 patients. Genes are ranked by the log2 fold change. (**G and H**) hPCLS have been infected for 24 h with Spn (10^3^ CFU) and co-treated with an IL-1β blocking antibody (10 µg/mL) (*N* = 3, duplicates). (**G**) Bacterial titers have been measured. (**H**) Reverse transcription-quantitative PCR has been performed on a panel of genes associated with IL-1β signaling for Spn infection. Statistical significance was established with a one-way analysis of variance (*N* = 3, each symbol represents one independent experiment). White boxes are the Spn-infected hPCLS, and gray boxes are co-treated with anti-IL-1β cocktail.

Lastly, we were interested in testing the specificity of the transcriptomic response of the PCLS model to a viral infection. Using a bacterial challenge with *S. pneumoniae* (serotype 3), we expected a distinct host response. As for the viral infection, bacteria penetrated the tissue to the center section in human PCLS (Fig. 6A). Twenty-four hours post-infection with *S. pneumoniae*, we observed a substantial increase in bacterial titers associated with the lung tissue for both hPCLS and mPCLS (Fig. 6B; Fig. S5A). In contrast to mouse *in vivo*, titers of Spn were much lower, potentially due to active bacterial clearance (Fig. S5A). The host response to Spn infection showed a higher donor variation in hPCLS and was generally less pronounced as for the viral challenge (Fig. 6C), with patient 1 not responding to the bacterial challenge despite robust bacterial replication. Since the same patient tissue reacted well to viral challenge (Fig. 5), we conclude this is a pathogen-specific effect. In contrast to the IAV response, the antibacterial host profile was, however, largely characterized by proinflammatory marker genes (e.g., IL1A, IL1B, TNF, IL6, and IL17A) and interleukin signaling in the Reactome analysis (Fig. 6D through F; Fig. S5H). The two murine systems showed a reasonable overlap in Spn-induced genes (Fig. S5C) and mirrored this NF-κB-driven proinflammatory response (NLRP3, IL1A, IL1B, and TNF), displaying markers of inflammasome activation (Fig. S5E through H). Lastly, we tested cell-to-cell communication in the human *ex vivo* model by blocking IL-1β signaling with a specific antibody. We compared the expression of IL-1β dependent genes after challenge with Spn in hPCLS. Without affecting the bacterial replication (Fig. 6G), the blocking of IL-1β signaling reduced the respective antimicrobial tissue response (Fig. 6H), confirming a robust cell-to-cell communication in this complex *ex vivo* model.

## DISCUSSION

Primary respiratory tract epithelial cells (e.g., human lung-on-a-chip models, lung organoids, or stratified airway epithelial cells [often used in air liquid interface]) have been shown superior to immortalized lung epithelial cells with regard to antiviral responses (26), notably with a broader ISG response. However, these models are mostly limited to the contribution of epithelial cells to lung infection biology. The PCLS model provides the cellular dimensionality of parental donor tissue while preserving its three-dimensional architecture. Unsurprisingly, it rapidly gained importance in biomedical studies targeting lung pathologies and pharmacological testing. mPCLS and hPCLS have been used to study the pathophysiology of inflammatory lung diseases (27, 28) and to test their response to disease-relevant proinflammatory stimuli (29). In respiratory infectious disease research, animal PCLS and hPCLS gain importance, for example, to assess the action of antimicrobials in a relevant organ context (30–32). Others used PCLS models before to assess the innate immune response to viral challenge (8, 33, 34) or to colonization with different mixes of commensal bacteria (mPCLS) (10). Two recent studies investigated viral replication and antiviral host responses in hPCLS by single-cell RNA-seq from a single human donor (35, 36). *Ex vivo* culture lung tissue from chronic obstructive pulmonary disease patients with terminal lung emphysema was used to characterize early antiviral and antibacterial responses, 24 h after IAV, *Pseudomonas aeruginosa*, or *Mycobacterium tuberculosis* (Bacillus Calmette-Guérin [BCG]) challenge, by single-cell RNA-seq and bulk RNA-seq (36). Importantly, the authors noted a high donor-dependent variation in host responses. They further stated that the pre-inflamed state of these tissues might not be fully representative of the antimicrobial response in healthy lung tissue. This could explain why only a limited number of IAV-upregulated genes were found as compared to our study. We provide here comprehensive transcriptome data in human and murine *ex vivo* tissue compared to *in vivo* host responses and complement these findings with a detailed characterization of the cellular composition of the two PCLS models. While not surprising, we show a clear contribution of the tissue resident memory cells to human host responses against IAV infection *ex vivo*. The production of IFNγ indicates a previous encounter with IAV, which would be supported by published serological data suggesting an exposure to IAV every 5–10 years (22). As for laboratory mice-derived PCLS, the biological differences to hPCLS in responsiveness to microbial infection likely stem from different genetics, metabolism, microbiome, and baseline immune status. The last point is of particular interest when studying infectious agents since many *in vivo* studies rely on immunologically naïve animals. Our data indicate that mPCLS from naïve mice rather poorly respond to IAV infection as compared to *in vivo* infections or hPCLS. In part, the lack of infiltrating immune cells into the PCLS model explains these differences, as well as the absence of tissue resident immune cells. Beyond this, infiltrating monocytes and neutrophils contribute substantially to the cytokine response and eventually immune pathology observed during a severe viral pneumonia (37). Exogenous addition of monocytes from blood or bone marrow might be a strategy to compensate for the absence of a connection to the supply with blood-derived immune cells to overcome the limited antiviral response. Our data suggest that bone marrow-derived macrophages could specifically restore the proinflammatory response to IAV when added to mPCLS. If these cells respond in a similar fashion as *in vivo* infiltrating cells during an infection is currently unclear. PCLS from animals with multiple IAV encounters could serve as a better model to mimic the immune history observed in human patients, who on average undergo an IAV infection every 5–10 years (22). Surprisingly, the innate antiviral host response did not differ in these animals. We did, however, find a modestly increased secretion of IFNγ, suggesting that tissue-resident T lymphocytes might contribute to the increased protection. Another striking difference between hPCLS and mPCLS was the low number of B lymphocytes in hPCLS, which was previously reported (38). This discrepancy is likely a species-specific phenomenon, since the donor lung tissue already shows a higher representation of B-lymphocytes in murine tissue (39, 40) than in human tissue (41).

These species-specific differences have to be taken into account when extrapolating from mouse to human data. Globally, we observed specific antiviral and antibacterial responses in both PCLS systems, suggesting a clear distinction between the two types of pathogens. This is also reflected in the alternative use of signaling pathways and downstream transcription factors driving host response programs. This is exemplified by the poor proinflammatory signaling following IAV infection, especially in the naïve murine *ex vivo* model. Curiously, NF-κB- and NOD2-driven genes are enriched in the host response of mPCLS to bacterial infection, ruling out an intrinsic deficiency in proinflammatory signaling and rather pointing toward a distinct upstream sensing of viral or bacterial pathogens. It must be noted that the timing (48 h IAV vs 24 h Spn) could in part explain these differences. Notably, a recent report indicated that PCLS undergo a spike of innate immune and inflammatory gene induction on the day of cutting, which wanes within 24 h (42). It is unclear how this mechanic induction of the innate immune response affects a subsequent challenge with viral or bacterial pathogens. The lack of IFNγ induction by IAV infection in mPCLS suggested that the early antiviral response in *ex vivo* tissues of naïve animals relies purely on a type I interferon response. In contrast, antibacterial/proinflammatory signaling after Spn challenge was readily detectable in mPCLS but less in hPCLS. This also suggests that mPCLS are not generally unresponsive to microbial exposure. In fact, an important contributor to the antibacterial host responses in mice is age. PCLS from aged mice display a stronger inflammatory reaction to bacterial PAMP than tissues from young animals (43). In hPCLS from aged patients, we detected a distinct proinflammatory host response after Spn colonization, as compared to the antiviral response. Globally, this response was less pronounced as the antiviral response despite robust bacterial replication. In fact, hPCLS from one human patient did not upregulate innate immune response genes after Spn challenge, but at the same time responded well to IAV, which suggests potentially an individual response based on previous exposure to pneumococci and potentially depending on patient-specific subsets of tissue-resident immune cells. Alternatively, tolerance mechanisms toward this common pathobiont in the upper respiratory tract of humans might play a role in dampening the antibacterial response. This could indicate that hPCLS could reflect the individual exposure history of a given patient and hence be used to study the basic cellular response in lung tissue based on immune history-shaped cell composition. Such a tool would be useful to predict innate responses in lung tissue of different age groups, for example, to novel influenza viruses, where preexisting immunity can play an important role to prevent mortality in critical target groups as reported for the 2009 H1N1 pandemic (44). IFI27, for example, was previously identified as a marker for IFN responses in peripheral immune cells (monocytes and conventional dendritic cells) of influenza virus-infected human patients (45), suggesting that some of these immune cells could reside in hPCLS. The shortage of human biopsy/autopsy material collected early during an IAV infection makes it difficult to conclusively answer these questions. On a more applied note, we think that hPCLS could be used to test antivirals or anti-inflammatory drugs in a realistic environment with additional viral restriction provided by the tissue resident immune cells and with an architecture and accessibility reflecting that of the native tissue.

### Limitations

A key component of antimicrobial responses is the dynamic infiltration of immune cells, which is currently not modeled in *ex vivo* tissues or other respiratory cell culture systems. In this regard, our *ex vivo* models only reflect very early antimicrobial host responses. Our data suggest that the addition to the mPCLS of bone marrow or peripheral blood-derived immune cells could enrich the host responses to viral and bacterial infection. A mechanistic explanation for the reduced viral titers in this approach or in the immune-experienced mPCLS model is currently elusive and remains a matter of ongoing investigations. The human *in vivo* transcriptional response was obtained from a single male patient deceased with influenza B-positive PCR. Hence, gender and age differences to our PCLS tissue donors could affect the transcriptional responses to IAV infection. Another important variable is the time between infection and tissue preparation for analysis. At 48 h, infection of *ex vivo* tissue reflects a very early host response, while *in vivo* the patients might not even be symptomatic at this stage. Human patients were mechanically ventilated, which could cause an additional stimulus absent in the *ex vivo* system. Larger post-mortem cohorts from influenza deceased patients will be required to address this limitation.

## MATERIALS AND METHODS

### PCLS generation

PCLS protocol was adapted from the literature (6, 33).

#### mPCLS

C57BL/6J mice (female, 7–10 weeks of age) were purchased from Charles River Laboratories (France) and housed at least 7 days under SPF conditions with a strict 12h/12h light/dark cycle and food and water *ad libitum*. Animals were euthanized by intraperitoneal injection of pentobarbital (150 µg/g, Esconarkon, Streuli Pharma, Uznach, Switzerland) when experimental or humane endpoints were reached. To collect lung tissues, the abdominal cavity and the thoracic cage were opened surgically. The inferior vena cava was nicked, and lung lavage was performed by phosphate-buffered saline (PBS) 1× injection into the heart right vena until the lung became white. Next, a catheter (22 G) was inserted in the trachea to perfuse the lungs with 2% low-melting-point agarose warmed to 37°C (Sigma-Aldrich, A9414, diluted in Dulbecco’s Modified Eagle Medium [DMEM]/Ham’s F12, Gibco). The mouse was cooled at 4°C for 10 min, and then the lung was excised and incubated 30 min at 4°C to let the agarose solidify. The lobes were dissociated, and murine PCLS (250 µm thickness) were cut with a Krumdieck tissue slicer (Alabama R&D, USA, MD600).

#### hPCLS

The use of human tissue was approved by the cantonal ethics committee of Geneva (CCER, project number 2022-01942). Lung tissues were provided by the thorax surgery unit of the University Hospital Geneva (Hôpitaux Universitaires de Genève, HUG). Tumor-free tissue from lung cancer resection surgeries of 26 patients was collected for this work. Patient data are indicated in Table S1. The lung segment was washed with PBS 1× and perfused with 2% low-melting-point agarose thanks to catheters (22 G). The solidification was ensured by incubating the tissue for 1 h 30 min at 4°C on ice. Using a coring press (Alabama R&D, USA MD500), tissue cylinders of 8 mm diameter were generated and transversally sliced into PCLS as described for mPCLS. PCLS were maintained in 500 µL DMEM/Ham’s F12 (Gibco, supplemented with 100 U/mL penicillin and 100 µg/mL streptomycin (Sigma-Aldrich, P0781) without fetal calf serum [FCS]) in 24-well plate at 37°C, 5% CO2. Low-melting agarose used for inflation was washed out by three sequential washes with warm PBS, and medium was changed after 2 h post-slicing. Then, lung slices were incubated at 37 °C, 5% CO₂ overnight (mouse and human) in complete DMEM to allow recovery before stimulation. Tissue integrity and absence of agarose was controlled with a brightfield microscope (Evos microscope system, Thermo Fisher). Only structurally intact slices with preserved alveolar architecture, visible airway epithelium, and consistent thickness were used. Slices showing hemorrhage or tissue collapse were excluded. For each experimental condition, one 250 µm PCLS was placed per well in a 24-well plate containing 500 µL of pre-warmed culture medium. For RNA extraction, two duplicates were pooled together.

### Bacteria

Spn (ATCC: [Klein] Chester 6303, serotype 3) was grown in Trypticase Soy Broth (Oxoid, UK, BO0369M) at 37°C with 5% CO_2_ in static culture up to 0.4 optical density (OD600 nm). PCLS infection was performed using 10^3^ CFU per well in the PCLS culture medium at 37°C, 5% CO_2_. The inoculum was removed after 4 h incubation, PCLS were washed two times with PBS 1×, and fresh culture medium was added. The infection was stopped 24 h post-inoculation. Supernatants and tissues were collected. Tissues were homogenized with a BeadBlaster homogenizer (Benchmark, D2400) using 1.4 mm ceramic beads to determine bacterial titers (Omni International, 19627). Serial dilutions were spotted on Trypticase Soy agar plates with 5% sheep blood (BioMerieux, M1006) at 37˚C with 5% CO2.

### Viruses

A/Netherlands/602/2009 (H1N1) (46) was kindly provided by Dr. Florian Krammer (Icahn School of Medicine at Mount Sinai, New York, NY, USA). PCLS were infected with 10^5^ PFU in 200 µL of PCLS medium per well. After 50 min of viral adsorption at 37°C, PCLS were washed with PBS 1× and incubated in fresh culture medium. Supernatants were collected at the indicated times post-infection, and viral titers were determined by plaque assay in Madin-Darby canine kidney (MDCK, ATCC) cells.

### Plaque assays

MDCK cells were grown in Dulbecco’s mModified Eagle medium (DMEM; Gibco) supplemented with 10% (vol/vol) heat-inactivated fetal bovine serum (Gibco) and 100 U/mL penicillin and 100 µg/mL streptomycin (Sigma-Aldrich, P0781). Cells (six-well plate format, 1.5 × 10^6^ cells/well) were infected with 10-fold serial dilutions in PBS 1× 0.2% bovine serum albumin (BSA) of the virus for 50 min at 37°C. Supernatants were replaced by fresh overlay medium supplemented with 1 mg/mL of N-tosyl-L-phenylalanine chloromethyl ketone-treated trypsin (Sigma) and 0.6% purified agar (Oxoid), and plates were incubated at 37°C 5% CO_2_. After 48 h, cells were fixed for 1 h at room temperature (RT) with 4% paraformaldehyde and the overlays were removed. Cells were stained using a solution of 16% methanol and crystal violet, and plaques were counted visually.

### *In vivo* infection

Mice were injected intraperitoneally with a mix of ketamine/xylazine (100 and 5 mg/kg, respectively) in 200 µL of sterile PBS. Upon reaching deep anesthesia, mice were inoculated with 40 µL of PBS, H1N1 virus (10^4^ PFU for RNA-seq or 10 PFU for the memory experiment) or *Spn* bacteria (10^6^ CFU) via the intranasal route. Lungs were collected after 24 h and 48 hpi for IAV, at 24 hpi for *Spn*.

### Immune cell complementation of mPCLS

Femurs and tibiae from 7- to 10-week-old female mice were harvested, and bone marrow was flushed using PBS 1× through the bones with a 25 G syringe. Cells were cultured in RPMI 1640 Glutamax medium supplemented with 10% (vol/vol) heat-inactivated fetal bovine serum (Gibco), 100 U/mL penicillin, 100 µg/mL streptomycin (Sigma-Aldrich, P0781), and 30 ng/mL of granulocyte-macrophage colony-stimulating factor (GM-CSF) for the BMDMs (Peprotech, ThermoFisher, 315-03-20). BMDMs were differentiated for 7 days prior to experimental use. For undifferentiated mixed bone marrow cells, complementation was performed immediately after an overnight incubation in complete medium without GM-CSF. A total of 2 × 10⁵ cells were added directly in immersion to PCLS following the influenza A virus (IAV) attachment step.

### RNA extraction and RT-qPCR

Tissue was stored at −80°C in RNA protect Tissue reagent (Qiagen, 76,104, Germany). PCLS were disrupted in the lysis buffer from the kit with 1.4 mm ceramic beads (Omni International, 19627) using a BeadBlaster homogenizer (Benchmark, D2400). Then, RNA extraction was performed with the NucleoSpin RNA XS kit (Macherey-Nagel, 740,902), following the manufacturer’s instructions. Elution was done in 30 µL of water. RNA concentration was estimated by Nanodrop. Two-hundred fifty nanograms was used for reverse transcription using the MMLV-RT (Invitrogen, 28025013). Gene expression was assessed by quantitative PCR using 2× KAPA SYBR FAST qPCR Master Mix-Universal (KAPA Biosystems, USA) and 10 µM of forward and reverse primers (Table S2). PCR was realized with CFX Connect Real-Time PCR Detection System (Bio-Rad) following this thermal cycling protocol: an initial denaturation step at 95 °C for 10 min, followed by 45 cycles of denaturation at 95 °C for 15 s, annealing/extension at 61.5 °C for 60 s, with a final melting curve step from 60°C to 95°C with 0.5°C increment. The relative gene expression was calculated with the ΔΔCt method, using *hprt* (murine tissue) or 18S rRNA (human tissue) genes for normalization.

For all lungs from mice or unprocessed lung biopsies from humans, total RNA was extracted using TRIzol (Ambion, Life Technologies) following manufacturer’s instructions.

### Lactate dehydrogenase (LDH) assay

To determine global cell suffering, lactate dehydrogenase (LDH) release was quantified daily into the PCLS supernatant using the colorimetric test CYQUANT LDH cytotoxicity assay kit (Invitrogen, C20300), according to the manufacturer’s recommendations.

### Cytokine release

IL-1β was quantified from PCLS culture supernatants using a BD OptEIA Human IL-1β ELISA Set II (557953) or Invitrogen Mouse IL-1β ELISA Kit (88-7013). Assays were performed on Nunc‐Immuno plates (Thermo Scientific) according to the manufacturer’s instructions. Captured cytokines were quantified against a serially diluted standard by reading absorbance (*A* 450 nm) values in a Thermo Scientific Multiskan GO. Analysis was done using the ELISA standard curve.

For the priming experiment, we quantified the release of one cytokine panel by multiplex assay using the LEGENDplex Mouse Inflammation Kit (Biolegend, 740446) and followed the manufacturer’s instructions.

### RNAscope

PCLS from mice and human were collected under sterile conditions, fixed in PBS 1× paraformaldehyde 4% solution overnight at 4°C and embedded in paraffin and sliced using a standard microtome (5 µm thickness). *In situ* detection of *Streptococcus pneumoniae* (RNAscope Probe-B-S. pneumoniae-SPN23F12870-C2, accession number: FM211187.1, nucleotides: 2–753) and IAV (RNAscope Probe V-Influenza A-H1N1–segment 5–NP-O8-C1, accession number: CY176945.1) was performed using the RNAscope 2.5 HD Assay (RED) (Advanced Cell Diagnostics) according to the manufacturer’s protocol. Briefly, tissue slices were deparaffinized by 1 h incubation at 60°C followed by four rounds in 100% xylol and four rounds in 100% ethanol solutions. An incubation with hydrogen peroxide was performed for 10 min, and target retrieval was achieved by incubating the slides with RNAscope 1× Target Retrieval Reagent for 45 min at 95°C. Tissue was permeabilized using RNAscope protease plus for 30 min (Advanced Cell Diagnostics). Probe hybridization was performed with a specific RNA probe for IAV or Spn for 2 h at 40°C. Signal amplification was achieved by incubating the tissues at 40°C with AMP solutions provided by the kit. Cyanine 3 and cyanine 5 were used as fluorophores, respectively, for IAV and Spn. Slides were counterstained with ProLong Diamond Antifade Mountant with DAPI (Invitrogen, P36962). Slides were visualized under Nikon A1r spectral microscope under a ×10/20 objective and processed using Zen Software.

### Transcriptomic analysis

RNA was extracted as described before at the replication peak of each pathogen (48 h for IAV, 24 h for Spn). RNA-seq (30 million reads per sample (triplicates for each murine condition, 5 patients samplings for hPCLS) was performed at the iGE3 Genomics Platform at the University. Analysis of these data was realized with the help of the Bioinformatics Support Platform (UNIGE). Libraries were prepared with the Illumina TruSeq HT Stranded mRNA protocol for PCLS and mouse *in vivo* lungs and with RNA exome-seq for human *in vivo* autopsy samples and corresponding control. The sequencing was done on Illumina NovaSeq 6000 sequencer. Sequenced reads were mapped with HISAT2 aligner either to the mouse genome GRCm39 (source: Ensembl 110) or to the human genome GRCh38.p14 (source: Ensembl 110). Read-count quantification was extracted with the method summarizeOverlaps() from R package GenomicAlignments. Normalization, differential gene expression analysis, and data visualization were conducted with the R programming language. Package DESeq2 was used to assess the statistical significance of differential gene expressions. More specifically, we conducted four independent DESeq2 analyses to take into consideration samples pairing: (ii) one with nine unpaired samples of the mouse *in vivo* conditions (mIV_mock, mIV_IAV, and mIV Spn), (ii) another with the 12 paired samples of the mouse PCLS conditions (mPCLS_mock, mPCLS_IAV, and mPCLS Spn), (iii) a last with 15 paired samples of the human PCLS conditions (hPCLS_mock, hPCLS_Spn, and hPCLS_IAV), and (iv) one with six unpaired samples of the human *in vivo* conditions (hIV_mock and hIV_Influenza). The details of the samples used in each set are given in Table S3. Pathway enrichment analysis was done under the R programming language with hypergeometric tests against Reactome.org database. Transcription factor enrichment analysis was realized using EnrichR (17–19).

### Flow cytometry

One day post-slicing, 14 PCLS per condition were washed with cold PBS 1× and pooled together. Cell dissociation was performed for 45 min at 37°C (agitation) with PBS + 5% fetal bovine serum (FBS) with 0.5 mg/mL collagenase D and 0.1 mg/mL DNase I. Cells were centrifuged for 5 min at 300 g. Red blood cell lysis was performed with ammonium–chloride–potassium buffer for 4 min at RT. The reaction was stopped with RPMI 10% FBS, and cells were spinned and washed in PBS.

Cells were stained 15 min at RT with Fixable Viability Dye (Invitrogen, 1:1,000 in PBS 1×), and Fc receptor (FcR) blocking was performed with 0.25 µg of TruStain FcX PLUS (Biolegend) in 100 µL of MACS before staining. Cells were then stained for 15 min on ice with surface marker antibodies resuspended in MACS buffer (PBS-0.5% BSA with 2mM EDTA) according to the panel. Antibody references are indicated in Table S4. Cells were fixed with 200 μL of IC Fixation Buffer (Thermo Fisher) for 30 min at RT. Samples were centrifuged at 450g at RT for 5 min. Cells were resuspended in an appropriate volume of MACS buffer, and the samples were run on LSRII Fortessa instrument (BD Biosciences).

### Cytokine blocking experiment

hPCLS were infected with 10^3^ CFU of Spn, after 4 h of attachment, PCLS were washed two times with PBS 1×, and culture medium containing 10 µg/mL antibody anti-IL-β (R&D Systems, MAB601, clone 2805) was added. Tissues and supernatants were collected 24 hpi.

### Antiviral treatment assay in hPCLS

hPCLS were infected with 10^5^ PFU of A/Netherlands/602/2009 (H1N1), after 1 h of adsorption, hPCLS were washed two times with PBS 1×, and fresh culture medium was added. Eight hours later, oseltamivir carboxylate (HY-13318, MCE,) was added at different concentrations: 2, 200, and 400 µM. Supernatants were collected 48 hpi, and titers were measured by plaque assay.

### Statistical analysis

Statistical analysis was performed using GraphPad Prism 9 or R, and the statistical tests applied as well as the number of biological and technical repeats are indicated in each respective figure legend.

## Data Availability

Sequencing data are available on GEO database with the following accession number: GSE306406. The code and software used in the analysis were packaged into a docker container made publicly available at https://github.com/BioinfoSupport/ngs (container), https://github.com/BioinfoSupport/rnaseq (mapping and quantification pipeline), and https://github.com/BioinfoSupport/genomes (for HISAT2 indexed genomes).

## SUPPLEMENTAL MATERIAL

Fig. S1 mbio.00056-26-s0001.pdf Viability of mPCLS and hPCLS in culture post-slicing.

Fig. S2 mbio.00056-26-s0002.pdf Flow cytometry gating strategy.

Fig. S3 mbio.00056-26-s0003.pdf Signaling activation in mouse *in vivo* and *ex vivo* 48 h after IAV infection.

Fig. S4 mbio.00056-26-s0004.pdf Immune cell complementation in IAV-infected mPCLS.

Fig. S5 mbio.00056-26-s0005.pdf *Spn* challenge in murine *in vivo* versus *ex vivo* system.

Supplemental legends mbio.00056-26-s0006.docx Descriptive legends for Fig. S1 to S5.

Table S1 mbio.00056-26-s0007.pdf Patient metadata.

Table S2 mbio.00056-26-s0008.pdf Oligonucleotides.

Table S3 mbio.00056-26-s0009.pdf RNAseq sample description.

Table S4 mbio.00056-26-s0010.pdf FACS reagents.

ASM does not own the copyrights to Supplemental Material that may be linked to, or accessed through, an article. The authors have granted ASM a non-exclusive, world-wide license to publish the Supplemental Material files. Please contact the corresponding author directly for reuse.
