# Supplementary figures and images for: Ex vivo modeling of lung tissue resident antimicrobial responses
Source: mBio. 2026 Apr 16;17(5):e00056-26. doi: 10.1128/mbio.00056-26 (PMC13170359; doi:10.1128/mbio.00056-26)

**A)**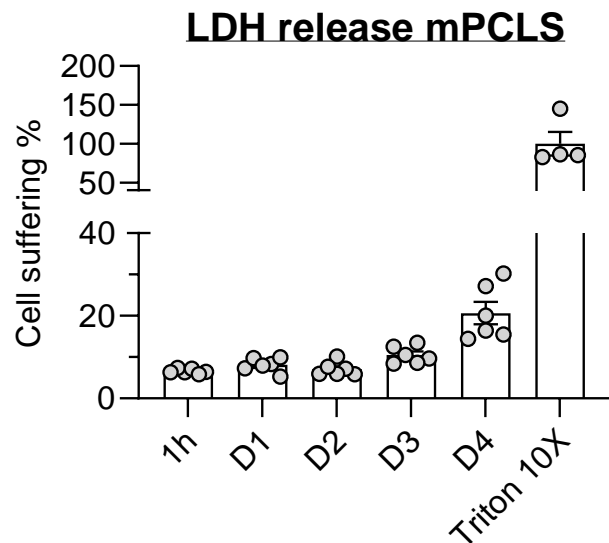**B)**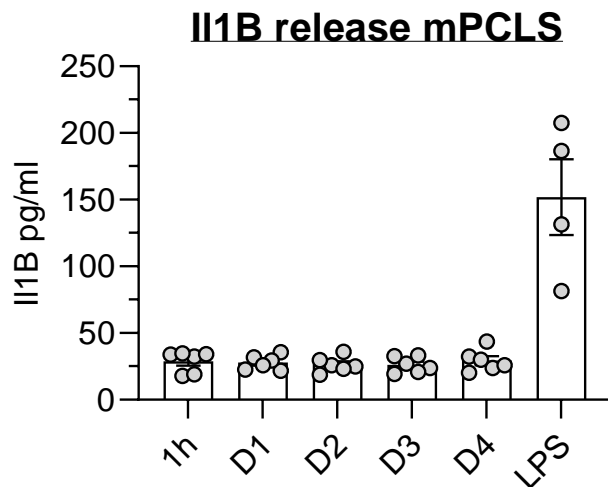**C)**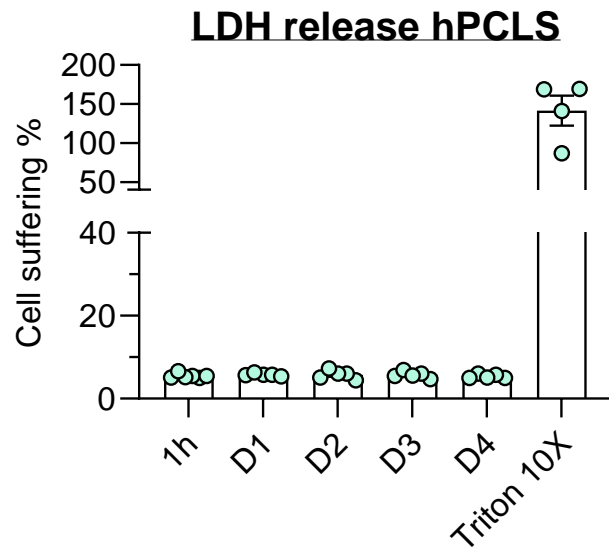**D)**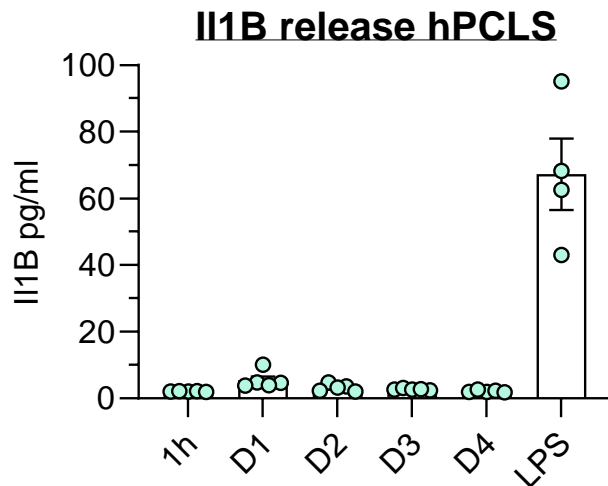

Supplement: Fig. S1 — Viability of mPCLS and hPCLS in culture post-slicing. [file mbio.00056-26-s0001.pdf]

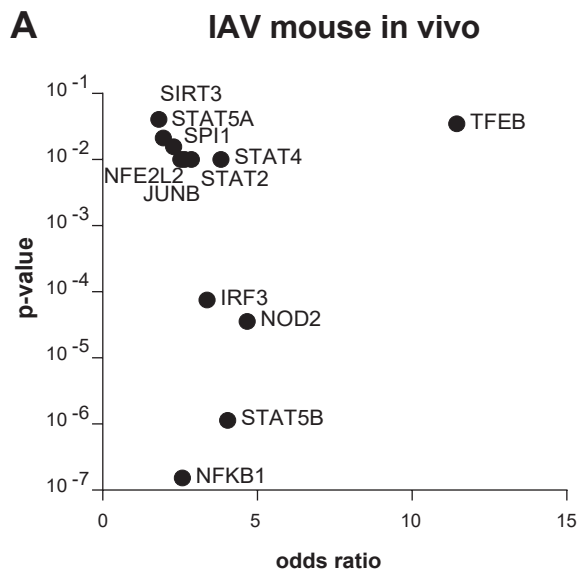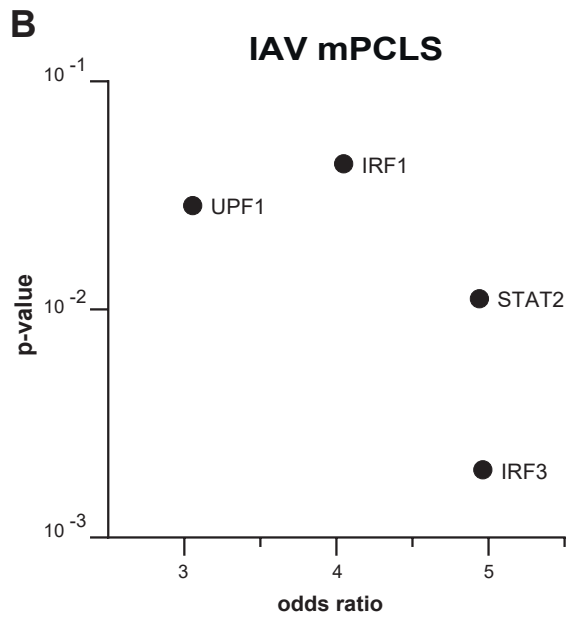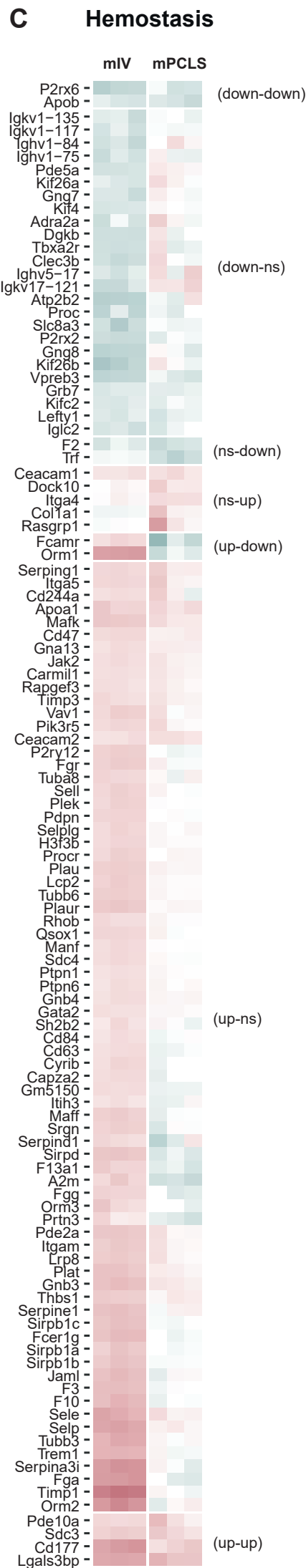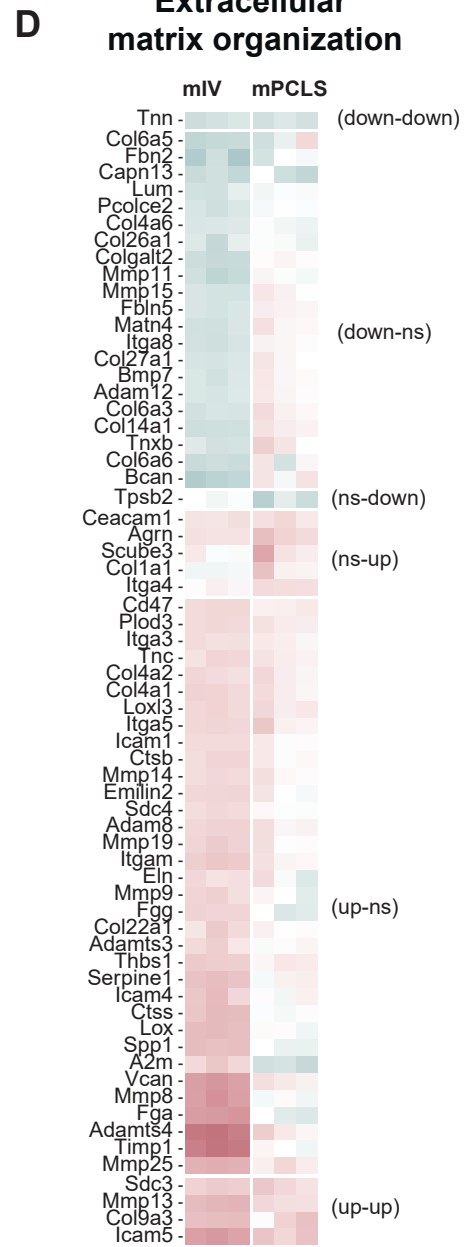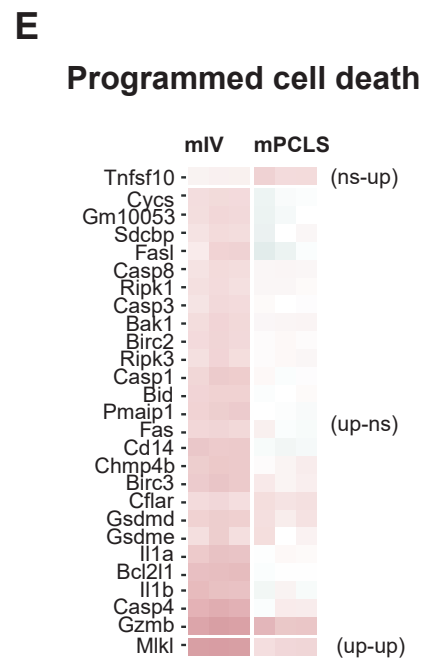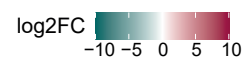

Supplement: Fig. S3 — Signaling activation in mouse in vivo and ex vivo 48 h after IAV infection. [file mbio.00056-26-s0003.pdf]

**A**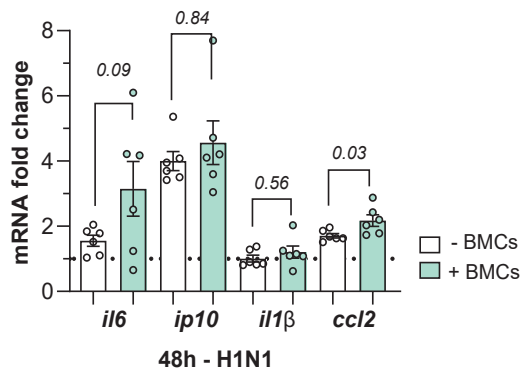**B**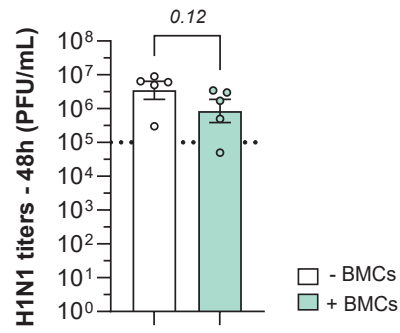**C**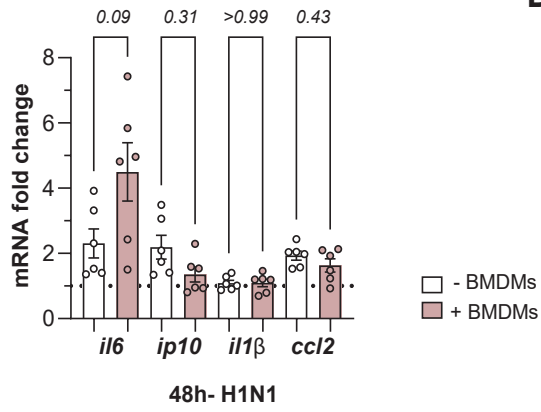**D**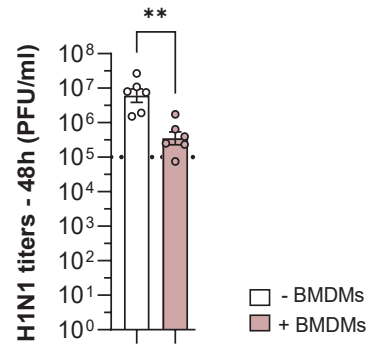

Supplement: Fig. S4 — Immune cell complementation in IAV-infected mPCLS. [file mbio.00056-26-s0004.pdf]

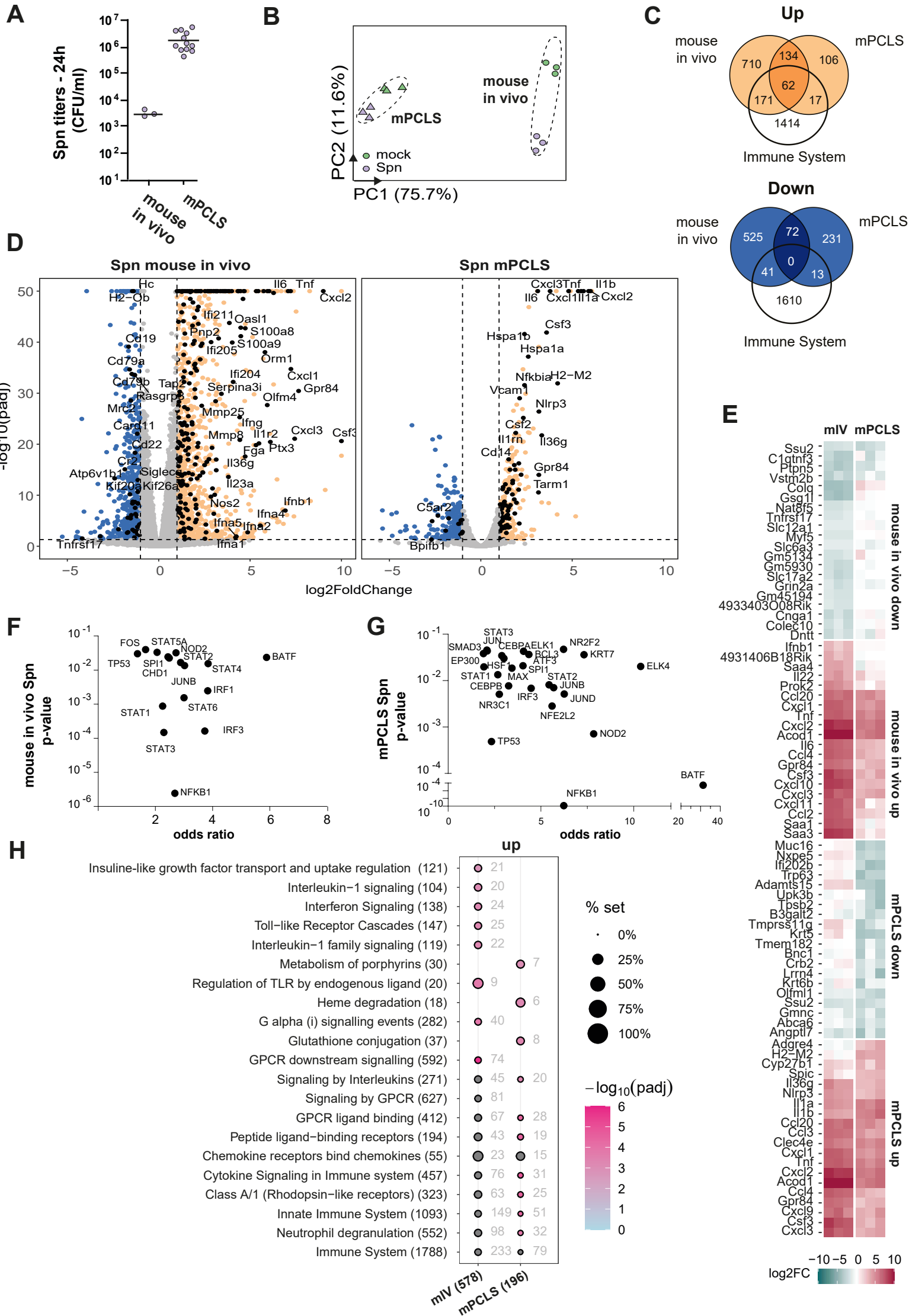

Supplement: Fig. S5 — Spn challenge in murine in vivo versus ex vivo system. [file mbio.00056-26-s0005.pdf]
