# Supplementary material for: Ex vivo modeling of lung tissue resident antimicrobial responses
Source: mBio. 2026 Apr 16;17(5):e00056-26. doi: 10.1128/mbio.00056-26 (PMC13170359; doi:10.1128/mbio.00056-26)
Supplement: Fig. S2 — Flow cytometry gating strategy. [file mbio.00056-26-s0002.pdf]

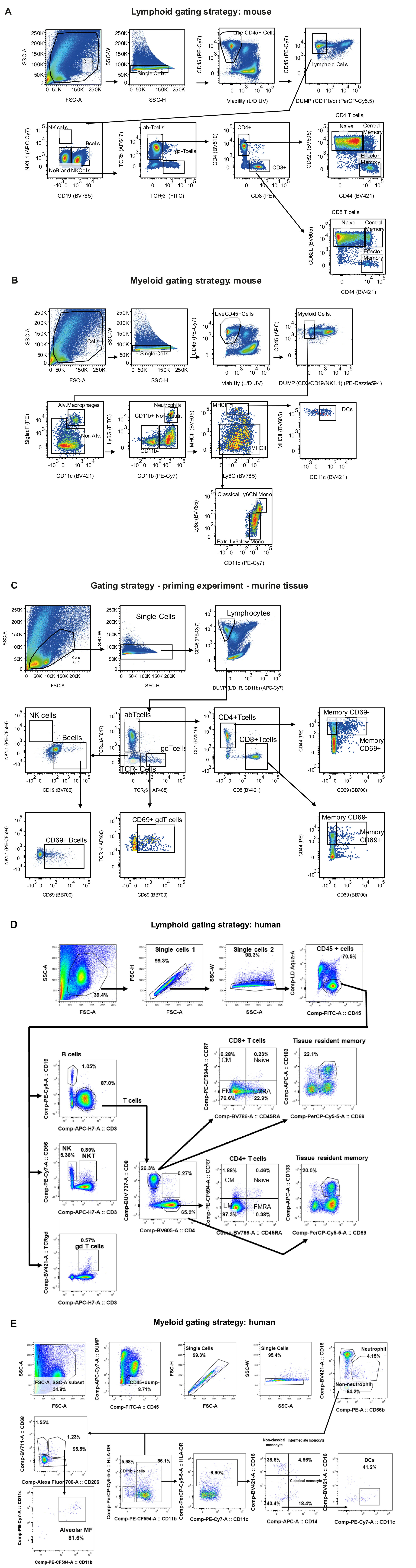
A
Lymphoid gating strategy: mouse
SSC-A
FSC-A
Cells
SSC-W
SSC-H
Single Cells
CD45 (PE-Cy7)
Live CD45+ Cells
Viability (L/D UV)
Lymphoid Cells
DUMP (CD11b/c) (PerCP-Cy5.5)
NK cells
Bcells
NoB and NKCells
NK1.1 (APC-Cy7)
CD19 (BV785)
ab-Tcells
gd-Tcells
TCRb (AF647)
TCRγδ (FITC)
CD4+
CD8+
CD4 (BV510)
CD8 (PE)
CD4 T cells
Naive
Central Memory
Effector Memory
CD62L (BV605)
CD44 (BV421)
CD8 T cells
B
Myeloid gating strategy: mouse
LiveCD45+Cells
Myeloid Cells.
CD45 (APC)
DUMP (CD3/CD19/NK1.1) (PE-Dazzle594)
Alv.Macrophages
Non Alv.
SiglecF (PE)
CD11c (BV421)
Neutrophils
CD11b+ Non-Neutr.
CD11b-
Ly6G (FITC)
CD11b (PE-Cy7)
MHCII hi
MHCII
MHCII (BV605)
Ly6C (BV785)
DCs
Classical Ly6Chi Mono
Patr. Ly6clow Mono
Ly6c (BV785)
C
Gating strategy - priming experiment - murine tissue
Cells
51.0
Single Cells
Lymphocytes
DUMP (L/D IR, CD11b) (APC-Cy7)
NK cells
Bcells
NK1.1 (PE-CF594)
CD19 (BV786)
abTcells
gdTcells
TCR- Cells
TCRαβ AF647
TCRγδ AF488
CD4+Tcells
CD8+Tcells
CD4 (BV510)
CD8 (BV421)
Memory CD69-
Memory CD69+
CD44 (PE)
CD69 (BB700)
CD69+ Bcells
CD69+ gdT cells
TCR γδ AF488
D
Lymphoid gating strategy: human
39.4%
Single cells 1
99.3%
Single cells 2
98.3%
CD45 + cells
70.5%
Comp-LD Aqua-A
Comp-FITC-A :: CD45
B cells
1.05%
87.0%
Comp-PE-Cy5-A :: CD19
Comp-APC-H7-A :: CD3
T cells
NK
5.36%
0.89%
NKT
Comp-PE-Cy7-A :: CD56
0.57%
gd T cells
Comp-BV421-A :: TCRgd
26.3%
0.27%
65.2%
Comp-BUV 737-A :: CD8
Comp-BV605-A :: CD4
CD8+ T cells
0.28%
CM
0.23%
Naive
EM
76.6%
EMRA
22.9%
Comp-PE-CF594-A :: CCR7
Comp-BV786-A :: CD45RA
Tissue resident memory
22.1%
Comp-APC-A :: CD103
Comp-PerCP-Cy5-5-A :: CD69
CD4+ T cells
1.88%
0.46%
97.3%
0.38%
20.0%
E
Myeloid gating strategy: human
FSC-A, SSC-A subset
34.8%
Comp-APC-Cy7-A :: DUMP
CD45+dump-
8.71%
Single Cells
99.3%
Single Cells
95.4%
Neutrophil
4.15%
Non-neutrophil
94.2%
Comp-BV421-A :: CD16
Comp-PE-A :: CD66b
1.55%
1.23%
95.5%
Comp-BV711-A :: CD68
Comp-Alexa Fluor 700-A :: CD206
Alveolar MF
81.6%
Comp-PE-Cy7-A :: CD11c
Comp-PE-CF594-A :: CD11b
5.98%
86.1%
CD11b - cells
Comp-PerCP-Cy5-5-A :: HLA-DR
6.90%
Non-classical monocyte
Intermediate monocyte
36.6%
4.66%
Classical monocyte
40.4%
18.4%
Comp-APC-A :: CD14
DCs
41.2%
