# Supplementary material for: Ex vivo modeling of lung tissue resident antimicrobial responses
Source: mBio. 2026 Apr 16;17(5):e00056-26. doi: 10.1128/mbio.00056-26 (PMC13170359; doi:10.1128/mbio.00056-26)
Supplement: Supplemental legends — Descriptive legends for Fig. S1 to S5. [file mbio.00056-26-s0006.docx]

**Supplementary figures:**

**Figure S1: Viability of mPCLS and hPCLS in culture post-slicing.**

Supernatants were collected from murine PCLS (gray) and human PCLS (green) at 1h post-slicing and for four days post-slicing. A-C) Cell death was measured by LDH release in mPCLS(A) and hPCLS(C). Positive control of cell death (100%) was realized with triton 10X treatment (N=3, duplicates). B-D) IL1β release was quantified by ELISA assay treatment in mPCLS (B) and hPCLS (D) (N=3, duplicates). Positive control of inflammation was realized by LPS treatment for 24h (0.5µg/ml).

**Figure S2: Flow cytometry gating strategy**

1. Flow cytometry gating strategy to describe lymphoid immune cells in murine PCLS
2. Flow cytometry gating strategy to describe myeloid immune cells in murine PCLS
3. Gating strategy used to describe resident memory lymphoid cells in murine PCLS
4. Flow cytometry gating strategy to describe lymphoid immune cells in human PCLS
5. Flow cytometry gating strategy to describe myeloid immune cells in human PCLS

**Figure S3: Signaling activation in mouse in vivo and ex vivo 48h after IAV infection**

Mice and mPCLS were respectively infected with 10^4^/10^5^ PFU of IAV (A/Netherlands/602/2009/H1N1) for 48h. Tissue was processed to bulk RNA sequencing and host response was analyzed using ‘DeSeq2’ package on R. Transcription factor enrichment analysis was conducted using EnrichR platform for IAV host responses in mouse in vivo (A) and ex vivo (B). Only transcription factors significantly enriched are indicated. Odds ratios are indicated in x-axis and p-value in y-axis. Hemostasis pathway (C), Extracellular matrix organization (D) and Programmed cell death (E) heatmaps in mouse system 48h after IAV infection

**Figure S4: Immune cell complementation in IAV-infected mPCLS.**

mPCLS were infected with 10^5^ PFU of IAV (A/Netherlands/602/2009/H1N1) for 48h. After the 1h viral adsorption step, mPCLS were washed with PBS 1X and new fresh medium was added. The medium was supplemented or not with 2. 10^5^ immune cells: A-B) a mixture of homologous undifferentiated bone marrow cells (BMCs) or C-D) autologous bone-marrow differentiated macrophages (BMDMs) directly with the mPCLS. At 48hpi, tissue and supernatants were collected to perform RT-qPCR on host inflammatory genes (A-C) and measure the viral replication by plaque assay (B-D). Statistical analysis was conducted using an unpaired non-parametric t-test (N=3, duplicates, **: p<0.01, *: p<0.05).

**Figure S5: *Spn* challenge in murine *in vivo versus ex vivo* system.**

Mice and mPCLS were respectively infected with 10^6^/10^3^ CFU of *Streptococcus pneumoniae* (Klein Chester ATCC 6303, serotype 3) for 24h. Tissue was processed to bulk RNA sequencing and host response was analyzed using ‘DeSeq2’ package on R.

1. Bacterial titers from lung tissue homogenates were measured at 24hpi (N=3 mice, N=6 mPCLS in duplicates).
2. Principal component analysis illustrates the transcriptional response of mock treated (green) and Spn-infected lung tissue (purple) *in vivo* and in mPCLS.
3. Venn diagrams of the commonly upregulated (orange) or downregulated (blue) genes in mouse in vivo and mPCLS infected with Spn. The white circle indicates the genes related to immune system pathways (using Reactome annotations).
4. Volcano plot showing differentially expressed genes between Spn infected and mock treated samples. Log2 fold-change induction is indicated in the x-axis, p-adjusted in the y-axis. Genes have been represented in: orange (upregulated), blue (downregulated), grey (not significant). Genes associated to immune system pathways are represented in black.
5. Heatmaps of the top 20 genes significantly upregulated (pink) and downregulated (blue) by Spn in mouse in vivo and mPCLS. Genes are ranked by the log2 Fold Change.

F-G) Transcription factor enrichment analysis was conducted using EnrichR platform for *Spn* host responses in mouse in vivo (F) and ex vivo (G). Only transcription factors significantly enriched are indicated. Odds ratios are indicated in x-axis and p-value in y-axis.

H) Pathway enrichment analysis was conducted using Reactome database (hypergeometric test). -log10 (p-adjusted) is given by a color scale. Coverage percentage of the pathway set is indicated by the size of the dot. Pathways are indicated with the total number of genes in brackets and the number of genes significantly regulated are indicated next to the dot.
