# Supplementary material for: Ex vivo modeling of lung tissue resident antimicrobial responses
Source: mBio. 2026 Apr 16;17(5):e00056-26. doi: 10.1128/mbio.00056-26 (PMC13170359; doi:10.1128/mbio.00056-26)
Supplement: Table S1 — Patient metadata. [file mbio.00056-26-s0007.pdf]

Table 1: Patient clinical data

N° Patient	Sex	Age	Pathology	Smoking	Location	Antecedents & Treatments	Experiment
**PCLS**							
2	M	70	Adenocarcinoma	Former (15 UPA)	Upper right	Atrial fibrillation under Clexane anticoagulant; hypertension; sleep apnoea syndrome; chronic normocytic normochromic anaemia	Kinetic IAV
3	F	56	Carcinoid	No	Middle right	Glaucoma; scoliosis	Kinetic IAV
4	F	83	Adenocarcinoma	Former	Upper right	Radiotherapy in 2020	RNAseq (sample 1)
6	M	78	Evolutive pulmonary nodule - left apex	No	Upper left	Melanoma resected in 2021	IF
7	M	57	Suspected lung nodule	No	Upper right	Hepatic steatosis; hypercholesterolemia; deep vein thrombosis; resected pituitary macroadenoma (2000, 2003); obesity ; sleep disorders. Treatments: Hydrocortison; Euthyrox; desmopressin; Xarelto; Beloc Zok; Aldactone; Lisitril; CalcimagonD3F; Crestor; Cipralex; Lipanthyl; Magnesium Diasporal; Pantoprazole; Sequase; Xanax; Zyloric	Kinetic IAV, IF
8	F	66	Suspected lung nodule	Active (80 UPA)	Upper left	COPD	Kinetic IAV, IF
9	F	61	Suspected lung nodule	Nd	Lower right	Lung adenocarcinoma (resected in 2018)	Kinetic IAV, IF
10	M	85	Suspected lung nodule	Active (70 UPA)	Upper left	Squamous cell carcinoma (lobectomy 2010); insulin-requiring diabetes	Kinetic IAV, RNAseq (sample 2)
11	F	57	Adenocarcinoma	Active (35 UPA)	Middle right	Arterial hypertension; dermatosis	Kinetic IAV, RNAseq (sample 3)
13	F	70	Suspected lung carcinoma	Active (50 UPA)	Lower left	Arterial Hypertension; hypercholesterolemia; BMI 33; Treatment: ANORO	Kinetic IAV, RNAseq (sample 4)
14	M	74	Suspected lung carcinoma	Former (40 UPA)	Upper left	Prostate adenocarcinoma (resected June 29, 2023); arterial hypertension treated with Amlodipine 5 mg; hypercholesterolemia treated with Ezetimib-Rosuvastatine	Kinetic IAV
15	M	61	Angiomyolipome	Nd	Upper right	Right nephrectomy; papillary thyroid carcinoma (resection) ; prostate adenoma	Kinetic IAV
29	F	65	Suspected lung nodule	Former (25 UPA)	Upper right	Carcinome canalaire du sein droit traité en 2019 (mastectomy) et 2020; cholescystectomy (2010)	LDH
30	F	57	Adenocarcinoma	Active (60 UPA)	Upper right	Colorectal adenocarcinoma (surgery 2024) - Toradol, Ondansétron	LDH
31	F	80	Suspected lung nodule	Active (60 UPA)	Upper left	Pulmonary emphysema; hypercholesterolemia; Symbicort Turbuhaler	LDH
43	M	74	Atypical carcinoid tumor	Former (70 UPA)	Lower left	COPD, arterial hypertension, type II diabetes, Treatments: diuretics, lipid-lowering drugs, anti-diabetics, Aspirine cardio	FACS
44	F	68	Suspected lung nodule	Former (70 UPA)	Upper left	COPD, lung adenocarcinoma (2020); arterial hypertension, Treatments: Amlodipine, Tobramycine, Aspirine cardio	FACS
45	M	73	Suspected lung nodule	Active (100 UPA)	Lower left	COPD, type 2 diabetes (without insulin), Treatments: Simcora; Pantoprazol; Candesartan; Sitagliptin	FACS
46	M	45	Suspected lung nodule	ND	Upper right	Dyslipidemia	Inhibitory test
47	M	51	Suspected lung nodule	Active (40 UPA)	Upper right	COPD, arterial hypertension; chronic rhinitis, Treatments: Candesartan; Wegovy; Montelukast; Symbicort Turbuhaler; Oxis; Kalcipos; cetirizine	Inhibitory test
49	M	78	Suspected lung nodule	ND	Upper right	-	Inhibitory test
58	M	73	Squamous cell carcinoma	Former (50UPA)	Lower right	Ischemic stroke, alcohol hepatitis (2012), arterial hypertension, dyslipidemia, non-insulin-dependent diabetes	RNAseq (sample 5)
68	M	83	Suspected lung nodule	Active (50 UPA)	Upper left	COPD, sleep apnea, arterial hypertension, obesity BMI 31	Antiviral test
69	F	66	Suspected relapse of adenocarcinoma	Active	Upper right	Former resection of lung adenocarcinoma in 2024, COPD, oesophagitis, obesity, arterial hypertension, cured hepatitis C (2022)	Antiviral test
70	F	67	Ground-glass nodules	Active (15 UPA)	Upper left	Arterial hypertension, sleep apnea, hepatic fibrosis	Antiviral test
**Samples for *in vivo* transcriptomic analysis**							
57	F	80	Suspected lung nodule	No	Upper right	-	Control RNAseq
58	M	73	Squamous cell carcinoma	Former (50UPA)	Lower right	Ischemic stroke, alcohol hepatitis (2012), arterial hypertension, dyslipidemia, non-insulin-dependent diabetes	Control RNAseq
59	F	71	Suspected lung nodule	Former (30 UPA)	Lower right	Monoclonal gammopathy (2012), vasculitis (13,03,2025), chronic rhinosinusitis, epicardial coronary disease, arterial hypertension, hypothyroidism	Control RNAseq
Lung biopsies were collected from a patient deceased naturally with recent influenza infection. This patient was first infected with influenza A one month before his death, then Influenza B diagnosis was confirmed two days before his decease. Biopsies were sampled in each lobe and tested by RT-PCR to detect the virus.							
AL12	M	88	No Ct	Former (5UPA)	Middle right	Antibiotics (iminipem, amikacin), antifungal (fluconazole), Oseltamivir 2 days before death, Avodart, Jardiance, gabapentine, beloc, crestor, torem, trajenta, Nexium, Mircera, Fenta, Lasix, rivotril	Influenza RNAseq
AL14			No Ct		Upper left		Influenza RNAseq
AL15			Ct 37,5		Lower left		Influenza RNAseq
