# Supplementary material for: Ex vivo modeling of lung tissue resident antimicrobial responses
Source: mBio. 2026 Apr 16;17(5):e00056-26. doi: 10.1128/mbio.00056-26 (PMC13170359; doi:10.1128/mbio.00056-26)
Supplement: Table S2 — Oligonucleotides. [file mbio.00056-26-s0008.pdf]

Table 2: Primers sequences

	gene	sequence 5'-3'	
**Murine genes**	*hprt*	F	CTGGTGAAAAGGACCTCTCGAAG
		R	CCAGTTTCACTAATGACACAAACG
	ifng	F	TGGAGGAACTGGCAAAAGGATG
		R	GGACCTGTGGGTTGTTGACCTCAAA
	ifna	F	TCAAAGGACTCATCTGCTGCTTG
		R	CCACCTGCTGCATCAGACAAC
	ifnl1	F	GTTCAAGTCTCTGTCCCCAAAA
		R	GTGGGAACTGCACCTCATGT
	stat3	F	CAATACCATTGACCTGCCGAT
		R	GAGCGACTCAAACTGCCCT
	ifnb	F	CAGCTCCAAGAAAGGACGAAC
		R	GGCAGTGTAACTCTTCTGCAT
	ip10	F	TTCACCATGTGCCATGCC
		R	GAACTGACGAGCCTGAGCTAGG
	cxcl9	F	TCAGCTCTGTGCACCTCCAG
		R	AACTCTCTTGTCCACACTCAC
	isg15	F	GGTGTCCGTGACTAACTCCAT
		R	TGGAAAGGGTAAGACCGTCCT
	tnfa	F	GACGTGGAACTGGCAGAAGAG
		R	TTGGTGGTTTGTGAGTGTGAG
	ccl2	F	ACCTGCTGCTACTCATTCACC
		R	TCAAAGGTGCTGAAGACCTTAG
	il1b	F	GCAACTGTTCCTGAACTCAACT
		R	ATCTTTTGGGGTCCGTCAACT
	il6	F	AAGCCAGAGTCCTTCAGAGAG
		R	TTGCCGAGTAGATCTCAAAGTG
**Virus**	*m1*	F	AGATGAGTCTTCTAACCGAGGTCG
		R	TGCAAAAACATCTTCAAGTCTCTG
**Human genes**	*18s*	F	GTAACCCGTTGAACCCCATT
		R	CCATCCAATCGGTAGTAGCG
	il1b	F	CTCGCCAGTGAAATGATGGCT
		R	GTCGGAGATTCGTAGCTGGAT
	nfkb1	F	AAGCAATTGAAGTGATCCAGGC
		R	ACCACTGGTCAGAGACTCGG
	nfkb2	F	ACCAGTGTCATTGAGCAGATAG
		R	ATGCATGGCTGAGTCTCCATG
	irak2	F	TCAAGTCCATGGAGCGGGTG
		R	TCTTACAGAAGCTGCCAAAGG
	il1a	F	TAGTAGCAACCAACGGGAAGG
		R	ATTATACTTTGATTGAGGGCGTC
	il6	F	AAGTCCTGATCCAGTTCCTGC
		R	TGCTACATTTGCCGAAGAGCC
	il8	F	TTTTGCCAAGGAGTGCTAAAGA
		R	AACCCTCTGCACCCAGTTTTC
	tnfa	F	CTTCTCGAACCCCGAGTCAGTTG
		R	TGAGGTACAGGCCCTCTGATG
