# Supplementary material for: Ex vivo modeling of lung tissue resident antimicrobial responses
Source: mBio. 2026 Apr 16;17(5):e00056-26. doi: 10.1128/mbio.00056-26 (PMC13170359; doi:10.1128/mbio.00056-26)
Supplement: Table S3 — RNAseq sample description. [file mbio.00056-26-s0009.pdf]

Table 3 : RNAseq samples description

sample	protocol	organism	model	treatment	specimen_id	experiment_id
HC01	rnaseq	mouse	in vivo	mock		mIV
HC02	rnaseq	mouse	in vivo	mock		mIV
HC03	rnaseq	mouse	in vivo	mock		mIV
HC04	rnaseq	mouse	in vivo	IAV		mIV
HC05	rnaseq	mouse	in vivo	IAV		mIV
HC06	rnaseq	mouse	in vivo	IAV		mIV
HC07	rnaseq	mouse	in vivo	Spn		mIV
HC08	rnaseq	mouse	in vivo	Spn		mIV
HC09	rnaseq	mouse	in vivo	Spn		mIV
HC10	rnaseq	mouse	pcls	mock	np6	mPCLS_IAV
HC11	rnaseq	mouse	pcls	IAV	np6	mPCLS_IAV
HC12	rnaseq	mouse	pcls	mock	np7	mPCLS_IAV
HC13	rnaseq	mouse	pcls	IAV	np7	mPCLS_IAV
HC14	rnaseq	mouse	pcls	mock	np8	mPCLS_IAV
HC15	rnaseq	mouse	pcls	IAV	np8	mPCLS_IAV
HC16	rnaseq	mouse	pcls	mock	np9	mPCLS_Spn
HC17	rnaseq	mouse	pcls	Spn	np9	mPCLS_Spn
HC18	rnaseq	mouse	pcls	mock	np10	mPCLS_Spn
HC19	rnaseq	mouse	pcls	Spn	np10	mPCLS_Spn
HC20	rnaseq	mouse	pcls	mock	np11	mPCLS_Spn
HC21	rnaseq	mouse	pcls	Spn	np11	mPCLS_Spn
HC22	rnaseq	human	pcls	mock	hp4	hPCLS
HC23	rnaseq	human	pcls	IAV	hp4	hPCLS
HC24	rnaseq	human	pcls	Spn	hp4	hPCLS
HC25	rnaseq	human	pcls	mock	hp10	hPCLS
HC26	rnaseq	human	pcls	IAV	hp10	hPCLS
HC27	rnaseq	human	pcls	Spn	hp10	hPCLS
HC28	rnaseq	human	pcls	mock	hp11	hPCLS
HC29	rnaseq	human	pcls	IAV	hp11	hPCLS
HC30	rnaseq	human	pcls	Spn	hp11	hPCLS
hp58_IAV	rnaseq	human	pcls	IAV	hp58	hPCLS
hp58_Spn	rnaseq	human	pcls	Spn	hp58	hPCLS
hp58_mock	rnaseq	human	pcls	mock	hp58	hPCLS
HC34	rnaseq	human	pcls	mock	hp13	hPCLS
HC35	rnaseq	human	pcls	IAV	hp13	hPCLS
HC36	rnaseq	human	pcls	Spn	hp13	hPCLS
A1L2	probe	human	in vivo	IAV		hIV
A1L4	probe	human	in vivo	IAV		hIV
A1L5	probe	human	in vivo	IAV		hIV
hp57_hIV	probe	human	in vivo	mock		hIV
hp58_hIV	probe	human	in vivo	mock		hIV
hp59_hIV	probe	human	in vivo	mock		hIV
