# Supplementary material for: Ex vivo modeling of lung tissue resident antimicrobial responses
Source: mBio. 2026 Apr 16;17(5):e00056-26. doi: 10.1128/mbio.00056-26 (PMC13170359; doi:10.1128/mbio.00056-26)
Supplement: Table S4 — FACS reagents. [file mbio.00056-26-s0010.pdf]

Table S4 : FACS reagents

Reagent	*Identifier*	*Source*
Collagenase, Type 4	LS004188	Worthington
DNAseI	10104159001	Roche
LIVE/DEAD™ Fixable Blue Dead Cell Stain Kit	L34961	Thermofisher
True-Stain Monocyte Blocker	426101	Biolegend
LIVE/DEAD™ Fixable Near-IR Dead Cell Stain Kit, for 633 or 635 nm excitation	L34975	Thermofisher
TruStain FcX™ PLUS (anti-mouse CD16/32)	156603	Biolegend
**Anti-mouse antibodies**		
APC anti-mouse CD45	103111	Biolegend
PE/Dazzle™ 594 anti-mouse CD19	115553	Biolegend
PE/Dazzle™ 594 anti-mouse CD3	100245	Biolegend
PE/Dazzle™ 594 anti-mouse NK-1.1	108747	Biolegend
PE anti-mouse CD170 (Siglec-F)	155505	Biolegend
PE/Cyanine7 anti-mouse/human CD11b	101215	Biolegend
FITC anti-mouse Ly-6G	127605	Biolegend
Brilliant Violet 605™ anti-mouse I-A/I-E	107639	Biolegend
Brilliant Violet 421™ anti-mouse CD11c	117329	Biolegend
Brilliant Violet 785™ anti-mouse Ly-6C	128041	Biolegend
PE/Cyanine7 anti-mouse CD45	103113	Biolegend
PerCP/Cyanine5.5 anti-mouse/human CD11b	101227	Biolegend
PerCP/Cyanine5.5 anti-mouse CD11c	117327	Biolegend
APC/Cyanine7 anti-mouse NK-1.1	108723	Biolegend
Brilliant Violet 785™ anti-mouse CD19	115543	Biolegend
Brilliant Violet 510™ anti-mouse CD4	100553	Biolegend
PE anti-mouse CD8a	100707	Biolegend
Brilliant Violet 605™ anti-mouse CD62L	104437	Biolegend
Brilliant Violet 421™ anti-mouse/human CD44	103039	Biolegend
Alexa Fluor® 647 anti-mouse TCR β chain Antibody	109217	Biolegend
Alexa Fluor® 488 anti-mouse TCR γ/δ Antibody	118127	Biolegend
BD Pharmingen™ Alexa Fluor® 700 Hamster Anti-Mouse CD69	561238	BD
BB700 Armenian Hamster Anti-Mouse CD69	566501	BD
PE anti-mouse/human CD44 Antibody	103023	Biolegend
Brilliant Violet 421™ anti-mouse CD8a Antibody	100737	Biolegend
APC/Cyanine7 anti-mouse/human CD11b	101225	Biolegend
**Anti-human antibodies**		
CD45 FITC	555482	BD
CD69 PerCPCy5.5	560738	BD
CD27 PE	302808	Biolegend
CCR7 Pe-Dazzle-594	353236	Biolegend
CD19 Pe-Cy5	15019942	Invitrogen
CD56 Pe-Cy7	557747	BD
CD103 APC	563883	BD
CD127 APC-R700	565185	BD
CD3 APC-Cy7	344818	Biolegend
TCRgd BV421	331218	Biolegend
CD4 BV605	562658	BD
CD45RA BV785	304140	Biolegend
CD8 BUV737	612754	BD
HLA-DR PerCP-Cy5.5	560652	BD
CD66b PE	561650	BD
CD11b PE-CF594	562399	BD
CD11c Pe-Cy7	301608	Biolegend
CD14 APC	555399	BD
CD206 AlexaFluor-700	321132	Biolegend
CD3 APC-Cy7	344818	Biolegend
CD19 APC-Cy7	363010	Biolegend
CD56 APC-Cy7	318332	Biolegend
CD16 BV421	562874	BD
CD15 BV605	564232	BD
CD68 BV711	333831	Biolegend
